# Effectiveness of lasers in managing dentine hypersensitivity: an umbrella review

**DOI:** 10.1007/s10103-025-04656-9

**Published:** 2025-10-24

**Authors:** Faraha Javed, Rahena Akhter, Vesna Miletic

**Affiliations:** 1 Dr. Ziauddin Ahmed Dental College, Aligarh Muslim University, Aligarh, India; 2 Faculty of Medicine and Health, Sydney Dental School, The University of Sydney, Sydney, Australia

**Keywords:** Dentin hypersensitivity, Pain, Laser therapy, Desensitizing agents, Review, JBI

## Abstract

To synthesize evidence from systematic reviews evaluating the effectiveness of laser therapy in the management of dentine hypersensitivity (DH). A comprehensive search was conducted across nine databases—PubMed, Cochrane Library, Medline, Scopus, Embase, Web of Science, DARE, PROSPERO, and ProQuest Dissertations & Theses—from inception to June 2025. This review followed a registered PROSPERO protocol and adhered to JBI and Cochrane methodological standards. Twenty-five systematic reviews were included. Methodological quality and risk of bias were assessed using the JBI checklist and the ROBIS tool. Overlap of primary studies was evaluated using citation matrices and the Corrected Covered Area (CCA) method. Laser therapy, either as a standalone intervention or combined with desensitizing agents, was consistently associated with short-term reduction in DH-related pain. Several high-quality reviews supported its effectiveness over placebo or no treatment. However, results were inconsistent when compared to other desensitizing agents. Only 7 reviews were rated as high quality, and most reviews had a high risk of bias, primarily due to lack of protocol registration, incomplete search strategies, and insufficient reporting of bias assessment. The CCA score of 3.9% indicated slight overlap among primary studies. Laser therapy appears to be effective for short-term relief of dentine hypersensitivity, but current evidence is limited by methodological weaknesses in existing reviews. Standardized clinical trial protocols, long-term follow-up data, and cost-effectiveness studies are essential to inform future clinical and policy decisions.

**Supplementary Information:**

The online version contains supplementary material available at 10.1007/s10103-025-04656-9.

## Introduction

Dentinal or Dentine hypersensitivity (DH) is characterized by a sharp, transient pain caused by a neural response to external stimuli (osmotic, thermal, chemical, or tactile) triggering fluid movement in the exposed dentinal tubules [1–3]. It is often associated with gingival recession, leading to discomfort and reduced oral health-related quality of life (OHRQoL) [2, 4, 5]. DH can arise from dentine erosion, periodontal disease or aggressive brushing [4, 6].

DH prevalence varies from 11.5% to 33.5%, due to differences in populations, study methods, and diagnostic criteria [5, 6]. The condition is typically assessed through clinical examinations, self-reported questionnaires, responses to specific stimuli, and the exclusion of other dental or periodontal issues [1, 5]. However, the inconsistency in diagnostic criteria highlights significant uncertainty and a lack of consensus among dental professionals regarding the diagnosis and management of DH [5].

DH management primarily focuses on two key approaches: blocking pain signal transmission or occluding the dentinal tubules to prevent fluid movement [7]. Initially, DH treatment usually involves the use of homemade remedies, such as dentifrices containing desensitizing agents. These agents include ions like sodium fluoride, strontium acetate, arginine, or calcium-carbonate-based products, which block the entrances of dentinal tubules [8, 9]. While effective for mild cases, these products generally provide gradual relief and often require consistent use over weeks to achieve noticeable results. Additionally, their effectiveness may diminish under acidic oral conditions or with excessive mastication forces [9, 10].

When over-the-counter products fail to deliver medium- or long-term relief, in-office treatments involve fluoride varnishes, adhesive resin sealants, and advanced desensitizers [7–9]. While they provide more immediate effects, their longevity may be compromised by oral conditions, daily habits, and patient compliance. Moreover, there is no universally accepted “gold standard” protocol or material for the treatment of DH, as the effectiveness of these methods varies widely among individuals [9]. Therefore, despite the availability of desensitizing toothpastes and other conventional agents, their limitations in durability and effectiveness highlight the continued need to evaluate advanced approaches such as laser therapy for managing DH.

A laser is a highly focused beam of coherent, monochromatic light (or other electromagnetic radiation) produced by specialized devices [1]. In recent decades, advancements in laser technology have expanded its applications in dentistry. Lasers are now utilized for caries prevention, teeth bleaching, cavity preparation [11], DH management [1, 2], growth modulation, and diagnostic procedures [12]. In soft tissue treatments, lasers have proven effective for wound healing, tissue removal, exposing impacted or partially erupted teeth, photodynamic therapy for malignancies, and photostimulation of herpetic lesions [11, 12].

Laser therapy for DH was first explored by Matsumoto et al. in the mid-1980s, marking a significant advancement in its management. Since then, laser therapy has been widely explored due to its potential to provide effective and long-lasting relief for patients with DH [1].

Laser therapy addresses this issue by sealing or modifying the dentinal tubules, thereby preventing fluid movement and subsequent stimulation [8, 9]. Scanning electron microscope (SEM) studies indicate that lasers achieve this by vaporizing, melting, or recrystallizing the mineral components of dentine, effectively occluding the tubules. Additionally, some lasers are thought to influence nerve activity directly, reducing pain through nerve desensitization [1].

High-power lasers such as Nd: YAG, erbium-doped yttrium aluminum garnet (Er: YAG), erbium-chromium-doped yttrium-scandium-gallium garnet (Er, Cr: YSGG), and CO_2_ are primarily used to obliterate dentinal tubules. These lasers generate thermal energy that alters the dentine surface, sealing the tubules and providing relief from hypersensitivity [1, 2, 4, 9].

Low-power lasers, including GaAlAs and He-Ne, are used for photobiomodulation (PBM). Unlike high-power lasers, PBM does not involve significant temperature changes that could harm the pulp or dentine. Instead, it acts on the nerve transmission within the dental pulp, providing pain relief without altering the structure of the dentine. In addition to reducing hypersensitivity, PBM has been associated with anti-inflammatory, analgesic, and tissue repair effects [2, 4, 9].

The application of lasers in managing DH has been supported by various studies [1, 2, 4, 7–9], which highlight their ability to reduce sensitivity by targeting both the structural and neural components of the condition. However, while several studies suggest that lasers may be more effective than other treatments in alleviating DH [13–15], others found no significant advantage over placebo [16]. Furthermore, laser therapy has been noted to have limitations, particularly in severe cases of DH [17], and concerns about potential adverse effects, such as thermal damage to dental pulp, have been raised [18, 19].

Despite the availability of various treatments, no consensus has been reached regarding their efficacy, and there remains no universally accepted protocol for DH management using laser therapy [1, 2]. Although some lasers, such as Nd: YAG and Er, Cr: YSGG, show promise by occluding dentinal tubules through superficial dentine melting, the long-term effectiveness of this approach remains controversial [4].

A previous umbrella review (Majidinia et al.) [20] concluded that laser therapy is effective in reducing dentin hypersensitivity compared with placebo or no treatment. However, it was limited to nine systematic reviews, excluded clinical trials, and searched only up to November 2022. The present umbrella review updates and extends the evidence base by including 25 systematic reviews from nine databases (including gray literature), covering studies up to June 2025, with a broader PICO framework by incorporating comparisons with other desensitizing agents and assessing outcomes beyond pain reduction, such as patient satisfaction, recurrence, cost-effectiveness, and safety.

This umbrella review (systematic review of systematic reviews, overview) aims to synthesize the available evidence, address conflicting findings, and provide a clearer understanding of the role of lasers in DH treatment. By evaluating the efficacy, safety, and long-term outcomes of laser therapy compared to traditional treatments, this review aims to establish a more informed basis for clinical decision-making and future studies. This umbrella review addressed the following research question: “In patients with DH, how effective is laser treatment compared to other treatment modalities or no treatment in reducing hypersensitivity and achieving additional clinical benefits?”.

## Methodology

The protocol was registered in PROSPERO (CRD42025645176) and adhered to the methodologies outlined by the Joanna Briggs Institute [21] and Cochrane [22], and was reported in accordance with the PRIOR (Preferred Reporting Items for Overviews of Reviews) checklist (Appendix A) [23].

### Search strategy

The search was conducted in PubMed, Cochrane Library, Medline, Scopus, Embase, Web of Science, DARE, PROSPERO, and ProQuest (Dissertations and Theses) from inception to 3rd February 2025. The search was updated on 22nd June 2025 to ensure the inclusion of the most recent relevant studies. We attempted to search OpenGrey, which was historically used to retrieve European grey literature but it has been decommissioned.

A two-phase search strategy was implemented by two reviewers (FJ, RA) independently to identify relevant studies. In the first phase, a focused search was conducted using specific keywords and MeSH terms combined with Boolean operators (“AND” and “OR”) including “laser,” combined with terms such as “dental,” “dentine,” “dentin,” or “dentinal,” alongside “sensitivity” or “hypersensitivity,” and “systematic review” or “meta-analysis”. These keywords were applied across the selected databases. In the second phase, a manual search was carried out by carefully reviewing the reference lists of all included systematic reviews to identify any additional relevant studies that may have been missed in the initial search. The search strategy included both peer-reviewed and grey literature sources. Only English-language publications were considered. Full search strings are provided in Appendix B.

### Eligibility criteria

The inclusion criteria for this review were based on the PICOS framework [24] and encompassed the following components: **Population (P)** included children and adults with DH; **Intervention (I)** was laser treatment; **Comparison (C)** involved other dental interventions such as topical desensitizing agents, fluoride treatments, placebo, or no treatment; **Outcomes (O)** focused on the reduction of DH symptoms as the primary outcome whilst the secondary outcomes included patient satisfaction, recurrence rates, cost-effectiveness, and safety (e.g., adverse effects of laser treatment); **Study types (S)** included systematic reviews and meta-analyses of randomized controlled trials (RCTs), cohort studies, or clinical trials. No restrictions were placed on the duration of follow-up. Both short-term and long-term outcomes were considered eligible. Only articles with full texts available were included in the review. If the full text was not accessible, attempts were made to contact the corresponding authors via email. If no response was received, the articles were excluded. There were no restrictions on the publication date of the included studies, but only studies published in English were eligible.

The exclusion criteria included reviews that did not conform to the criteria for systematic reviews i.e. used a clearly stated set of objectives with pre-defined eligibility criteria, an explicit and reproducible methodology, a systematic search to identify all relevant studies, an assessment of the validity of findings, and a systematic presentation and synthesis of the characteristics and findings of the included studies.

### Study screening and selection

The search results were imported into Covidence (Covidence systematic review software, Veritas Health Innovation, Melbourne, Australia), where duplicate records were identified and removed before proceeding to screening. After the initial automated process, a careful manual review was performed to detect and eliminate any duplicates that may have been overlooked, taking into account slight differences in author names, publication information, or content descriptions. Title/abstract and full-text screening were performed independently by two reviewers (FJ and RA) according to predefined criteria to ensure unbiased and thorough selection of relevant studies. Any discrepancies or disagreements between the reviewers at either stage were resolved through discussion with a third reviewer (VM) until consensus was reached.

The process was carefully documented, and the number of studies included and excluded at each stage, along with reasons for exclusion, were recorded in a PRISMA flow diagram to maintain transparency and reproducibility in the study selection.

### Data extraction

The review adhered to the Joanna Briggs Institute (JBI) methodology for umbrella reviews, and the JBI Data Extraction Tool for Systematic Reviews and Research Syntheses (Appendix C) was used to guide this process.

Data extraction was performed independently by two reviewers (FJ and RA) using a pre-defined template. Extracted information included the author and year of publication, objectives, participant characteristics, number of patients or teeth involved, description of the intervention or phenomenon of interest, search details and keywords used, sources searched, range (years) of included studies, number of studies included, types of studies, country of origin of the included studies, appraisal and appraisal instruments used, appraisal ratings, analysis and method of analysis, outcomes assessed, results and significance, heterogeneity among studies, and reviewer comments. The scope of evidence and adverse events assessed in the systematic reviews were summarized narratively, with key details presented in data tables. Adverse events were specifically noted in the comments section of the JBI checklist during quality appraisal. Any discrepancies were resolved through discussion with a third reviewer (VM).

### Overlap of primary studies

To assess the extent of overlap among primary studies included in systematic reviews, citation matrices were constructed and “Corrected Covered Areas” (CCAs) were calculated. The CCA quantifies overlap by dividing the number of repeated occurrences of an “index” publication (the first appearance of a primary study) across other reviews by the product of “index” publications and reviews, minus the number of “index” publications. Measuring overlap is crucial in overviews of systematic reviews to prevent bias from double-counting primary studies, which can misrepresent the strength and reliability of the evidence. This method of quantifying and validating overlap using CCAs was proposed by Pieper et al. (2014) [25]. Covered Area (CA) is calculated as **N** divided by the product of **r** and **c**

$$\:CA=\frac{N}{rc}$$

while the Corrected Covered Area (CCA) is calculated as

$$\:CCA=\frac{(N-r)}{(rc-r)}$$

where **N** is the total number of included publications—counted by the number of ‘X’s marked in the citation matrix (Table 1); **r** is the number of “index” publications (rows), and **c** is the number of reviews (columns). A score (equalling % values) of 0–5 indicates slight overlap; 6–10, moderate; 11–15, high; and greater than 15, very high overlap [25].

**Table 1:** Citation matrix and calculation formulae

	Systematic review 1	Systematic review 2	Systematic review 3	Systematic review 4	Systematic review 5
Primary study 1	X				X
Primary study 2	X		X		
Primary study 3		X	X	X	
Primary study 4			X	X	
Primary study 5	X	X			X

Example based on Table 1.

- CA = 12/25 = 0.48 = 48%
- CCA= (12 − 5)/(5*5–5) = 7/20 = 0.35 = 35%

### Assessment of methodological quality/critical appraisal

The JBI critical appraisal tool for Systematic Reviews and Research Syntheses (Appendix D), was used by two independent reviewers (FJ and VM) to assess the quality of the included studies. The appraisal was conducted only at the systematic review level; no independent evaluation of the primary studies was performed. The tool consists of 11 key questions designed to guide the assessment of systematic reviews or meta-analyses. Each question is answered with “yes,” “no,” “unclear,” or “not applicable” (NA), the latter being used only in rare cases.

The questions evaluate important aspects such as whether the review question was clearly stated, the appropriateness of inclusion criteria, the adequacy of the search strategy and sources, the methods used for critical appraisal, data extraction, and synthesis of studies, as well as the assessment of publication bias. Additionally, the tool assesses whether recommendations for practice and research directions are supported by the evidence. This structured appraisal ensures that all relevant dimensions of review quality are considered and documented. These individual questions are considered together to provide an overall appraisal of each review, traditionally categorized as include, exclude, or seek further information. However, for this study, we adapted the appraisal ratings to “low”, “moderate”, or “high” quality, similar to the grading system used in GRADE analysis, to better reflect the strength of evidence and guide interpretation. Differences in scoring were settled by consensus of the two reviewers. The overall quality rating for each study was determined based on the number of “no” and “unclear” responses in the JBI critical appraisal checklist. Specifically:

- High quality: 0 “no” and ≤ 2 “unclear” responses.
- Moderate quality: 1–2 “no” responses or 3–4 “unclear” responses.
- Low quality: ≥3 “no” responses or ≥ 5 “unclear” responses.

### Risk of bias assessment

Risk of bias (RoB) was assessed by two reviewers using the ROBIS tool [26]. Following discussion and calibration, RoB initial rating was performed by FJ, and then checked by VM by analysing sections pertaining to specific signalling questions in each systematic review. Any discrepancies were resolved through consensus. ROBIS assessments in Phase 2 (bias in the review process) and 3 (overall RoB rating) were performed as Phase 1 is optional (assessing relevance). Phase 1 was not included as a similar relevance assessment was performed during critical appraisal phase, i.e. the JBI Critical Appraisal Checklist starts with an assessment of a review question.

Four domains were evaluated in the ROBIS Phase 2: (1) study eligibility criteria; (2) identification and selection of studies; (3) data collection and study appraisal and (4) synthesis and findings. The first 3 domains contain 5 signalling questions each whilst domain 4 contain 6 signalling questions. Each signalling question could be answered as “yes”, “probably yes”, “probably no”, “no” or “no information”. The analysed reviews were assigned an RoB rating for each domain as well as the overall RoB in the ROBIS Phase 3 as per the following classification:

- low risk of bias – all signalling questions answered with a “yes” or “probably yes”
- unclear risk of bias – 1 signalling question answered with a “no”, “probably no” or “no information”
- high risk of bias – more than 1 signalling question answered with a “no”, “probably no” or “no information”

### Synthesis methods

The results from the included systematic reviews were synthesized narratively, based on the data and conclusions reported by these reviews. Data extraction, quality assessment, and citation mapping were conducted systematically using Excel spreadsheets, allowing for organized evaluation and comparison of the included studies. A narrative synthesis was chosen due to the variability in review scopes, methodologies, and reported outcomes, which precluded statistical pooling. Exploration of heterogeneity was limited to the information provided within the systematic reviews, describing any potential causes of variation such as differences in populations or interventions as noted by the original authors. No additional sensitivity analyses were performed at the overview level; the robustness of findings was interpreted based on the assessments conducted within the included reviews.

## Results

### Search results

**Fig. 1:**
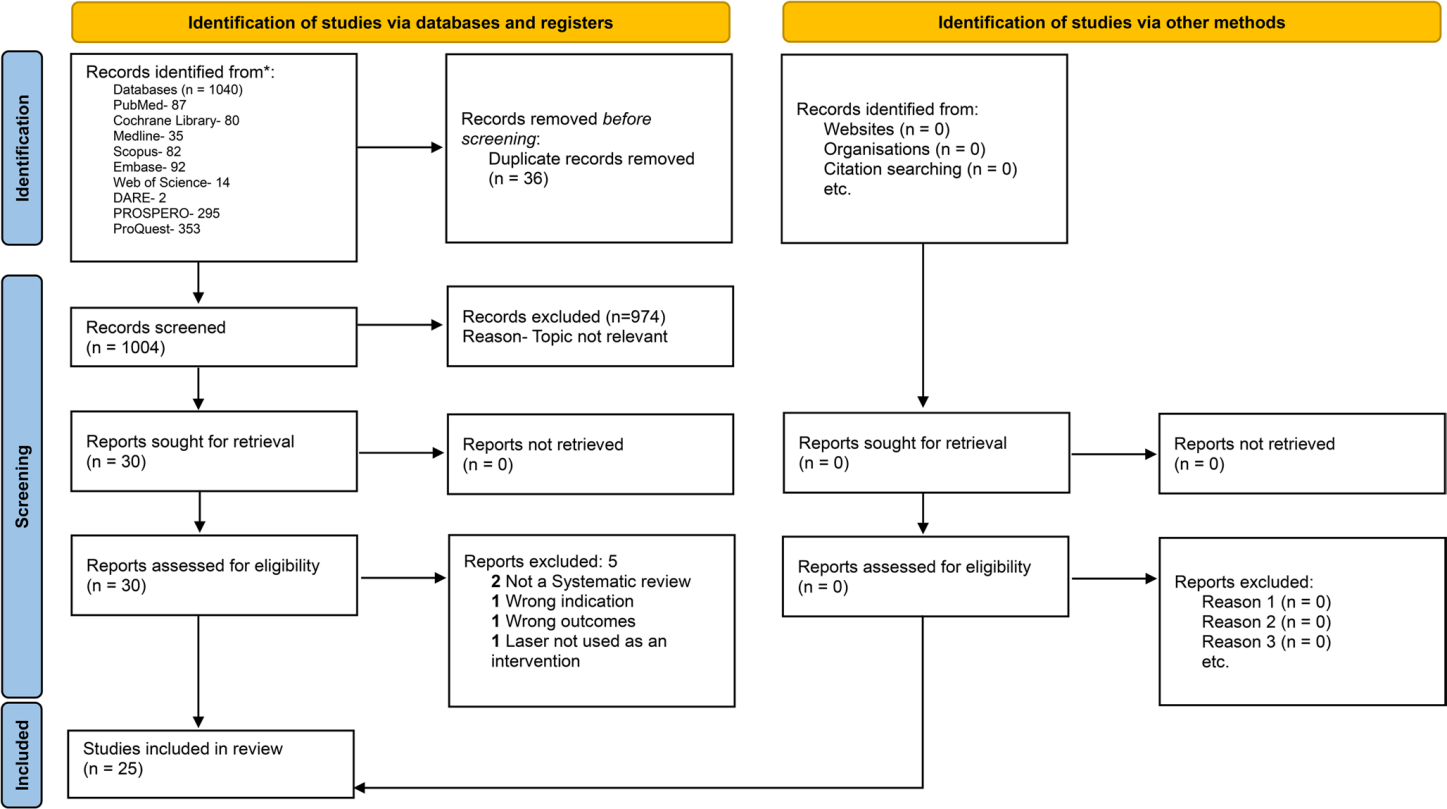
PRISMA flowchart of the article selection process

Study selection process is presented in the PRISMA flow diagram (Fig. 1). Initially, 1040 publications were identified. 36 duplicates were removed. The remaining 1004 publications were screened at the title and abstract level. 974 publications were removed as they pertained to the topics that did not match the area of interest. 30 publications were assessed for eligibility and 5 were excluded for the following reasons: 2 were not systematic reviews, 1 had the wrong indication, 1 assessed the wrong outcomes, and 1 did not use laser as an intervention. The remaining 25 publications were included in the review and data extraction.

### Characteristics of systematic reviews

The details of the analysed systematic reviews are presented in Tables 2 and 3. The spatial distribution of countries of origin of primary studies that were included in the systematic reviews was reported in 16 out of 25 reviews. Most primary studies originated from Brazil [1, 3, 8–10, 27–36], Turkey [1, 3, 8, 10, 27, 29–34, 36], and Iran [1, 3, 8, 10, 27, 30–32, 34–36]. The temporal analysis showed that more than half of the analysed reviews, 14 out of 25, were published after 2020 [1, 3, 4, 7, 8, 10, 27, 28, 37–40], 6 studies were published in the period 2015–2019 [2, 9, 31, 32, 34, 41] and 5 studies were published in the period 2010–2014 [29, 30, 33, 35, 42].

No specific settings of primary studies were mentioned in the majority of systematic reviews. Where specified, university setting was the predominant site of clinical trials [1, 8, 9, 37, 42] whilst non-specific single- or multi-centre studies were reported in one publication [41]. Majority of systematic reviews focused on RCTs, whilst a few reviews included other types of clinical trials, such as prospective clinical trial [7], single-arm trials [4], interventional studies [43] and unspecified in vivo studies [34, 38]. Follow-up periods varied considerably, with the longest follow-ups being 18 months [3, 10, 40], 24 months [40], and 25 months [41].

Majority of systematic reviews included patients without a pre-determined age or adult patients who experienced DH [3, 4, 7–10, 27–43] whilst a few studies specifically included patients younger than 18 years of age [1, 2] or patients with a specific condition or post-treatment associated with dentine sensitivity, such as MIH [40] or tooth bleaching [28]. Generally, the number of patients included in primary studies was reported whilst the number of teeth was less frequently mentioned or was mentioned partially depending on the availability of these data in primary studies. More than 16,334 patients and 29,659 teeth were included in the analysed systematic reviews. Neither patient nor teeth numbers were reported in 2 systematic reviews [28, 31].

Three distinct approaches could be observed in analysed systematic reviews: (1) various laser treatments compared to a variety of other desensitizing agents [4, 9, 28, 29, 34, 37, 43]; (2) various laser treatments compared to a specific control group (one desensitizing agent, placebo laser or no treatment) [1, 3, 7, 27, 31, 32, 35, 36, 41, 42]; (3) specific laser treatment compared to a variety of control groups (various desensitizing agents, placebo or no treatment) [8, 10, 38, 39]. In a few studies, laser combined with an additional desensitizing agent was tested [2, 30, 33, 40].

Pain intensity changes were determined using mostly VAS which was reported in the majority of analysed systematic reviews with or without other evaluation methods. Other methods were numerical rating scale (NRS) [3, 34, 36], verbal rating scale (VRS) [3, 28, 29, 31, 33, 34, 39, 42], VAS + plaque index [34], Yeaple probe [8, 39, 41], air stimulation and pressure [8], Modified VAS (MVAS) [28], 14-item Oral Health Impact Profile (OHIP-14) [40], the face scale [36], or the Uchida scale [4, 29, 34, 36, 38], numeric rating scale [4, 9, 31], Laser Doppler Flowmetry (LDF) [39], air-sensitivity scale (0 to 3) [1], evaporative stimulation method [2], pain scale (3/4 degrees) [4], and Schiff scale [41].

Where adverse effects were mentioned in analysed systematic reviews, no such effects were reported [1, 8, 9, 27–29, 33, 35, 43], except for one review with one primary study reporting pain immediately after treatment in two patients [42] and another review with two primary studies reporting mild discomfort, pain or tooth sensitivity to laser or other desensitizing agents [36].

In terms of critical appraisal of primary studies, majority of systematic reviews used at least a RoB tool, whilst 5 did not specify any critical appraisal [9, 30, 33, 34, 38]. Additionally, quality of evidence was assessed using the GRADE tool in 5 studies [1, 7, 31, 32, 36]. In terms of data analysis, meta-analysis was performed in 10 out of 25 studies [1, 4, 10, 27, 31–33, 36, 37, 42]. In studies with no meta-analysis, heterogeneity among analysed primary studies was highlighted in several studies [2, 8, 9, 28–30, 35, 41]. Moreover, high heterogeneity was reported in studies where meta-analysis was performed, with I² >60% [36] or even I² >80% [4, 10, 27, 31, 33, 36, 37, 42].

**Table 2:** Characteristics of analysed systematic reviews – aim, search strategy, primary sources

Author/Year	Objectives	Participants (Characteristics/total no.)	Setting/Context	Description of intervention/phenomenon of interest	Search details/ Keywords	Sources searched	Range(years)of included studies	Number of studies included	Types of studies included	Country of origin of included studies
Abdelkarim-Elafifi et al/2022 [3]	To describe parameters used with 808- to 980-nm wavelength diode lasers for managing dentin hypersensitivity and analyze their results.	Patients with DH, VAS values ≥ 2, aged > 18 years/398 patients/approx. 845 teeth (6/11 mentioned teeth)	NA	High- or low-intensity diode laser therapy alone or in combination with desensitizing agent vs. “Lack of diode laser therapy” or placebo (if applicable)	Articles published from 2009 until 2020, in English/dentin hypersensitivity, diode laser, GaAlAs laser, LLLT or low-level light	MEDLINE (PubMed, Cochrane)	2009–2020	11	RCTs	Brazil, Iran, Spain, Turkey, Italy, India,
Albar NH/2022 [7]	Systematically evaluate the efficacy of laser desensitization compared to GLUMA desensitizer in patients with DH for 6 months.	Patients with DH, no predetermined age/205 patients/894 teeth	NA	Laser vs. GLUMA desensitizing agent	Search performed on March 5, 2022, in English/laser Gluma desensitization	PubMed, Scopus, Web of Science, Grey literature search on Google Scholar	2009–2018	8	RCTs and PCTs with a minimum of 6-month follow-up	NA
AlHabdan and AlAhmari/2022 [8]	To assess the efficacy of Er, Cr: YSGG lasers in reducing dentine hypersensitivity (DH)	Patients with DH, aged 18 years/199 patients/approx. 542 teeth (6/7 mentioned teeth)	University setting	Er, Cr: YSGG laser vs. other dentine desensitizer agent/no treatment	Search performed up to March 2022, in English/laser therapy, phototherapy, dentin sensitivity or hypersensitivity, Er, Cr: YSGG	MEDLINE, Scopus, ProQuest, LILACS, EBSCO	2011–2021	7	RCTs	Iran, Brazil, Turkey, USA
Baghani and Karrabi/2022 [10]	To compare the air blast and tactile tests for assessment of the efficacy Nd: YAG laser therapy versus non-laser treatments for DH in short-term and long-term follow-ups.	Patients with DH, no predetermined age/606 patients/642 teeth	NA	Nd: YAG laser vs. non-laser treatment modality such as toothpaste, gel, mouthwash etc.	Articles published until March 10, 2021, in English/Dentin sensitivity or hypersensitivity, Nd: YAG Laser or neodymium-doped yttrium aluminum garnet laser	MEDLINE via PubMed, Cochrane, Scopus, Embase, Science Direct	2005–2019	9	RCTs	India, Turkey, Iran, Brazil, Lebanon, China
Bellal et al/2021 [27]	To compare immediate and 1-month post-treatment VAS scores of near-infrared laser group and placebo/no treatment group in patients with dentine hypersensitivity	Patients with DH, aged > 18 years/216 patients/approx. 671 teeth (5/6 mentioned teeth)	NA	Near-infrared lasers vs. placebo/no treatment group	Articles published between January 2000 and February 2021, in English/dentin sensitivity or hypersensitivity, low-level laser therapy or LLLT, photomodulation, Nd: YAG, laser diode laser, RCT, placebo	MEDLINE, PubMed, Cochrane, Science Direct, ProQuest, Open, Google scholar, Manual search, Grey Literature	2002–2019	6	RCTs (parallel and split-mouth study designs)	Norway, Iran, Brazil, Turkey,
Carneiro et al/2022 [28]	Assess effectiveness of low-level laser therapy (LLLT) in preventing tooth sensitivity after tooth whitening.	Patients who underwent DB, aged > 18 years/no. of patients or teeth not specified	NA	Low-level laser therapy/photobiomodulation vs. placebo or other treatment	Search performed on 4th May 2021, no language or publication dates restriction/dentine sensitivity or hypersensitivity, teeth whitening, bleaching, low level light therapy, photobiomodulation, LLLT	MEDLINE via PubMed, Cochrane, Scopus, Embase, Web of Science, SciELO, VHL Regional Portal, ClinicalTrials.gov, Manual search, Grey Literature	2016–2020	6	RCTs	Brazil
Cerqueira et al/2025 [40]	To assess the effectiveness of laser therapy, alone and in combination with other strategies, for DH in teeth affected by MIH.	Patients with MIH, no predetermined age range/612 patients	NA	Laser therapy, alone and in combination with other strategies	Search performed in March 2024/Dentin sensitivity, Hypomineralization, Laser therapy, Molar incisor hypomineralization or hypersensitivity or MIH, Dental enamel hypoplasia	PubMed, Scopus, Embase, Web of Science	2019–2023	7	RCTs	NA
Chen et al/2025 [36]	To determine whether lasers or topical desensitizing agents are more effective in managing dentin hypersensitivity (DH).	Patients with DH, no predetermined age/approx 286 patients (15/25 mentioned patients)/approx 1148 teeth (18/25 mentioned teeth, one study used " jaw quadrant” as a unit)	NA	Laser treatment vs. topical desensitizing agents	Search performed in December 2024/“dentin hypersensitivity,” “laser,” “varnishes,” “fluorides,” “topical,” “Gluma,” “dentin bonding,” “dentin desensitizer,” “potassium nitrate,” “saltpetre,” “potassium nitrate monohydrate,” “oxalate,” and “ethane dioic acid”	MEDLINE (PubMed), Cochrane, Scopus, Embase, Web of Science, ClinicalTrials.gov	2003–2024	25	RCTs	Brazil, China, India, Turkey, Lebanon, Iran, Thailand
He et al/2011 [29]	To systematically evaluate existing evidence to verify whether laser therapy provided a better performance compared to other desensitizing agents for treating dentine hypersensitivity.	Patients with DH, no predetermined age/234 patients	NA	Laser treatment vs. other topical desensitizing agents (fluoride varnish, dentin bonding agents, NK 10% gel, 3% potassium oxalate gel, 2% NaF, 5% NaF Varnish, Dentin Protector)	Articles published from 1970–2010, in English/dentin sensitivity’, ‘dentine sensitivity’, ‘dentinal sensitivity’, ‘dentin hypersensitivity’, ‘dentine hypersensitivity’, ‘dentinal hypersensitivity, ‘lasers’, ‘laser’.	MEDLINE, Cochrane Central Register of Controlled Trials (CENTRAL), Cochrane Oral Health Group’s Trials Register, National Research Register, Embase, Manual search	2002–2009	8	RCTs	Turkey, Spain, Brazil, Thailand, India, Germany
Hu et al/2019 [31]	To evaluate the immediate and long-term desensitizing effect of lasers in reducing dentine hypersensitivity (DH) compared with negative controls.	Patient with DH, no predetermined age/no. of patients or teeth not specified	NA	Laser treatment vs. placebo or no treatment controls	Search performed up to June 8, 2018, in English and Chinese/lasers, dentine hypersensitivity	PubMed, CENTRAL (Cochrane Library), Embase, Web of Science, China National Knowledge Infrastructure, Chinese Biomedical Literature Database, Manual search	1994–2018	22	RCTs	Turkey, Brazil, China, Iran, Spain, Australia, Norway, Germany, Korea, India
Kong et al/2019 [32]	To compare different lasers, placebo, and no treatment in the treatment of DH over different time periods.	Patients with DH, no predetermined age/786 patients	NA	Laser treatment (GaAlAs, Nd: YAG, Er: YAG, Er, Cr: YSGG, CO2), vs. placebo and no treatment.	Search performed on December 11, 2018, in English/lasers, dentine hypersensitivity	PubMed, CENTRAL (Cochrane Library), Embase, Web of Science, ProQuest Dissertation Abstracts, the International Clinical Trials Registry Platform, a thesis database, ClinicalTrials.gov, Manual search, Grey Literature	2002–2018	11	RCTs	Norway, Iran, Brazil, Turkey,
Lestari et al/2023 [39]	To compare the efficacy of Nd: YAG laser to other chemical agents in the treatment of dentin hypersensitivity	Patients with DH, no predetermined age/159 patients/approx. 402 teeth (5/6 mentioned teeth)	NA	Nd: YAG Laser vs. desensitizing agents	Publication period between 2012 and 2022, in English/Dentin Hypersensitivity, dentin hypersensitive, tooth sensitivity, dentin hypersensitive treatment, Laser, Nd: YAG, NdYAG, dentin desensitizing agents	PubMed, EbscoHost, ProQuest, Google Scholar	2012–2020	6	RCTs	NA
Lin et al/2013 [33]	To compare the effectiveness of different in-office desensitizing treatments in resolving dentin hypersensitivity.	Patients with DH, no predetermined age/approx. 2298 patients (34/40 mentioned patients)/approx. 857 teeth (7/40 mentioned teeth)	NA	group I: placebo; group II: physical occlusion of dentinal tubules; group III: chemical occlusion of dentinal tubules; group IV: nerve desensitization; group V: photobiomodulating action (Laser therapy); and group VI: combined treatment (any combination of groups II–V)	Search performed in December 2011, no language restrictions/“(dentin* OR tooth OR teeth) AND (hypersensit* OR desensiti* OR desensitize*), limited to “clinical trials” and “humans”;	MEDLINE (via PubMed), Cochrane Central Register of Controlled Trials (CENTRAL), Web of Science, Ovid, Science Direct, Manual search	1979–2012	40	RCTs (split-mouth and parallel trials)	NY, UK, Italy, USA, Norway, Germany, Brazil, Spain, China, Turkey, Greece, Thailand, India, Peru,
Machado et al/2018 [9]	To assess the effectiveness of PBM in the treatment for DH.	Patients with DH, aged > 18 years/75 patients/159 teeth	Universities	Low-power laser vs. other in-office treatments, placebo, or no treatment	Search performed in October 2016, no language restrictions/(laser low level OR laser low power OR diode laser OR 660–790 nm OR 780 nm) AND (dentin hypersensitivity OR dentin sensitivity OR dental pain OR dental sensitivity).	MEDLINE (via PubMed), Manual search	2004–2015	3	RCTs	Brazil
Mahdian et al/2021 [1]	To assess the effects of in-office lasers versus placebo laser, placebo agents or no treatment for relieving pain of dentinal hypersensitivity.	Patients with DH, aged above 12 years/936 patients/2296 teeth	University based-21 Multicenter study-1 Unclear-1	Laser treatment vs. placebo or no treatment	Search performed in October 2020, no language or publication date restriction/lasers, dentin sensitivity, hypersensitivity	Cochrane Oral Health’s Trials Register, the Cochrane Central Register of Controlled Trials (CENTRAL), MEDLINE Ovid, Embase Ovid, CINAHL EBSCO, LILACS BIREME Virtual Health Library, Web of Science, ZETOC, ClinicalTrials.gov, World Health Organization International Clinical Trials Registry Platform, Manual search, OpenGrey	1994–2020	23	RCTs	Brazil, Turkey, India, Australia, Norway, Spain, South Korea, Iran
Marto et al/2019 [2]	To compare the treatments used to treat dentin hypersensitivity (DH), based on its efficacy and effect duration	Patients with DH, aged 16–65 years/5366 patients/approx 9167 teeth (38/73 mentioned teeth)	NA	desensitizing products (Potassium nitrate, Potassium chloride, Potassium fluoride, Sodium fluoride (NaF), Stannous fluoride, Amine fluoride, Strontium acetate, Oxalates, Iontophoresis, Arginine, Glutaraldehyde + HEMA, Chlorhexidine, Strontium Chloride, hydroxyapatite, Herbal, Ozone, Composite resins, Adhesives systems, Glass ionomer cements, Dentin sealants, Laser, Potassium Citrate, HEMA, Others) and placebo	Publications between 1 January 2008 and 14 November 2018, in English, Portuguese or Spanish/dentin sensitivity, treatment, dentin desensitizing agents,	MEDLINE (through PubMed), Cochrane, Embase, Clinical Trials databases	2009–2018	73	RCTs	NA
Mohammadian et al/2025 [43]	To evaluate and compare the effectiveness of NaF varnish, diode laser therapy (DL), and their combination in reducing DH.	Patients with DH, no predetermined age/129 patients/882 teeth	NA	sodium fluoride varnish, diode laser, or their combination vs. placebo or comparisons between the interventions	Publication up to May 2024, in English/[(“fluoride varnish” OR “NaF varnish” OR “sodium fluoride” OR “diode laser” OR “sodium monofluorophosphate”) AND “dentin hypersensitivity”]	PubMed, Scopus, Web of Science, Google Scholar, Manual search	2016–2022	3	Interventional comparative study, Interventional study, RCT-split mouth	Saudi Arabia, India
Oliveira da Rosa et al/2013 [30]	To analyze the clinical effectiveness of current desensitizer with at least 3 months of follow-up	Patients with DH, no predetermined age/949 patients/3039 teeth	NA	Laser, Gluma desensitizer, Cervitec, Oxa-Gel, iontophoresis	Search performed in August 2012, in English, Portuguese or Spanish/dentin or dental sensitivity or hypersensitivity, desensitizing dentin, dentin desensitizing agents, teeth or dental desensitizer	MEDLINE(PubMed), Cochrane, Scopus, Embase, Web of Science, LILACS, IBECS, Scielo	2002–2012	17	RCTs	Brazil, Iran, India, Germany, Turkey, Norway, Netherlands
Pion et al/2023 [4]	To assess efficiency of laser therapy in the treatment of DH	Patients with DH, no predetermined age/approx. 4611 teeth (31/34 mentioned teeth)	NA	Laser treatment with or without another desensitizing agent	Search performed in April 2020, in English/laser, photodynamic therapy, dentin sensitivity, hypersensitivity	PubMed (MEDLINE)	1993–2019	34	RCTs (Split-mouth, parallel group), single-arm trials	NA
Rezazadeh et al/2019 [34]	To assess efficiency of various types of lasers for prevention and treatment of DH	Patients with DH, no predetermined age/approx. 1343 patients (36/39 mentioned patients)/approx.2892 teeth (24/39 mentioned teeth)	NA	Group 1: investigated laser application as a preventive procedure. Group 2: compared laser with placebo. Group 3: compared laser with desensitizing agents. Group 4: different types of lasers	Publications between January 2007 to December 2016/dentin or dentinal sensitivity, dentine hypersensitivity, lasers	MEDLINE, Scopus	2007–2016	39	RCTs and in vivo studies	Iran, UK, Korea, Turkey, India, Brazil, South Korea, Sudan, Italy, Germany, Macedonia, Spain, Thailand
Sgolastra et al/2011 [35]	To assess effectiveness of laser therapy compared with placebo laser therapy in the treatment of DH. The secondary aim was to assess laser treatment safety	Patients with DH, aged > 18 years/56 patients	NA	Mid- or low-power laser treatment vs. placebo	Search performed in August 2010, no language restrictions/dental OR dentin* OR tooth OR teeth OR cervic* OR cemen*) AND (sensitiv* OR hypersensitiv*) AND laser	MEDLINE (via PubMed), Science Direct Cochrane Clinical Trials Register, Cochrane Reviews, Web of Science, LILACS, International Association of Dental Research (IADR) Abstracts and Current Contents, Manual search	2002–2009	3	RCTs	Iran, Norway, Brazil
Sgolastra et al/2013 [42]	To assess the efficacy of lasers, stratified according to laser type, on changes in pain level, when compared with a placebo or no treatment. The secondary aim was to assess the safety of the laser application and the occurrence of adverse events.	Patients with DH, aged > 18 years/437 patients	University − 9 Both university and private clinics − 1 Unclear − 4	Laser treatment vs. placebo or no treatment	Search performed in February 10, 2013, no language or year restrictions/NA	MEDLINE, Cochrane Controlled Clinical Trial Register, Cochrane Database of Systematic Reviews, Scopus, DARE, CINAHL, Science Direct, Manual search	1994–2012	13	RCTs	NA
Shakeel et al/2022 [38]	To appraise the effect of GaAlAs lasers in the treatment of DH	Patients with DH, no predetermined age/150 patients/612 teeth	NA	GaAlAs laser treatment vs. placebo, NaF, dentin bonding agent	In English/GaAlAs Lasers, dentin hypersensitivity	PubMed, Cochrane, Scopus, Embase, Ovid MEDLINE, Science Direct, Wiley online library, Grey Literature	2003–2017	6	In-vivo studies	NA
West et al/2015 [41]	To evaluate the effectiveness of self- administered and/or professionally applied treatment modalities for the reduction in pain from dentine hypersensitivity.	Patients with DH, no predetermined age/12,568 patients	Majority were single center, few were multicenter and one was two-center	Nd: Yag laser, a laser-emitting toothbrush and GaAIAs laser treatment vs. placebo or fluoride paste	Search performed from 14–21 July 2014, in English/“hypersensitivity” OR “sensitivity” AND “dentin” AND one of the following treatments: arginine, NovaMin, oxalates, potassium, PVM copolymer, stannous, strontium, casein, Gluma, laser, Seal and Protect, Duraphat, Bifluorid, resin, varnish and glass ionomer.	PubMed, MEDLINE, Cochrane Database Trials Register, Manual search	1984–2014	105 (8 on lasers)	RCTs (two parallel design and six split-mouth)	NA
Zhou et al/2021 [37]	To compare the effectiveness of lasers and topical desensitizing agent treatments for DH	Patients with DH and with postoperative hypersensitivity, no predetermined age/1053 patients	All studies were conducted at dental college, except one which was at military medical academy	Laser treatment vs. other topical desensitizing agents (MI Varnish, Dentin bonding agent, Sodium Fluoride varnish, Novamine paste, Potassium oxalate gel, Gluma desnsitizer, Arginine Calcium carbonate)	Search performed in August 2019, no language restriction/keywords not specified	PubMed, Cochrane Central Register of Controlled Trials (CENTRAL), Embase, Web of Knowledge, Manual search	2005–2019	13	RCTs or CCTs	NA

*RCT *randomized clinical trial, *PCT* prospective clinical trials, *CCT* controlled clinical trial, *DH* dentine hypersensitivity, *NA* not available. At least 16,334 patients and 29,659 teeth

RCT- randomized clinical trial, PCT- prospective clinical trials, CCT- controlled clinical trial, DH- Dentine hypersensitivity, NA- Not available. At least 16,334 patients and 29,659 teeth.

**Table 3:** Characteristics of analysed systematic reviews - critical appraisal, analyses and main findings

Author/Year	Critical appraisal	Appraisal rating	Meta-analysis	Methodof analysis (refersto meta-analytical approach)	Outcomeassessed	Results/Significance	Heterogeneity	Comments/additional observations
Abdelkarim-Elafifi et al/2022 [3]	Risk of bias (Cochrane Collaboration tool)	NA	No	NA	VAS, VRS, NRS	Among the laser groups found in the reviewed papers, the baseline VAS ranged between 2.3 and 8.8. These values decreased after laser application to 0.45–3.7, with all results being statistically significant. All of the included studies showed a statistically significant improvement in treatment outcomes among the laser groups.	NA	Follow up periods- 12/24 weeks, 1/2/3/6/18 months. Heterogeneity in analyzed studies related to laser parameters, application techniques, and the number of sessions. Further RCTs in DH patients are recommended, using various diode laser wavelengths while keeping other parameters constant (e.g., energy density, evaluation scale, sessions, application points, emission mode, and exposure time) to identify the most effective infrared wavelength.
Albar NH/2022 [7]	Risk of Bias- ROB 2 for RCTs and ROBINS-I for non-randomized studies; Quality of Evidence (GRADE tool)	Based on GRADE, the overall quality of the evidence in this study was low. Based on ROB 2, four studies demonstrated an overall high risk of bias, and three studies demonstrated some concerns. Based on ROBINS-I, one study showed a lower risk of bias except for missing outcome data (some concerns)	No	NA	VAS	All the included studies showed improved results in treating DH from the baseline values to the post-treatment values. The immediate effect of pain relief was higher with GLUMA. The long-term analgesic effect was greater for laser therapy. No statistically significant changes were observed between low-level and high-level laser therapy.	NA	Follow-up period- Immediately to 6 months. Indication of university setting and indication of GLUMA and laser combination, no evidence offered.
AlHabdan and AlAhmari/2022 [8]	ROB 2 (Cochrane risk-of-bias tool for randomized trials)	low risk of bias, all included studies presented an appropriate randomization process, outcome measurement, and selection of the reported result.	No due to high heterogeneity	NA	VAS, precalibrated Yeaple probe, air stimulation and pressure	All the included studies reported that the use of the Er, Cr: YSGG laser decreased the pain level and dentine hypersensitivity.	NA	Adverse effects included-none found. Heterogeneity and small sample size highlighted and recommended long-term RCTs and large sample size Included studies demonstrated heterogeneity in study design, laser parameters, duration of application, stimulation method, and follow-up times.
Baghani and Karrabi/2022 [10]	Risk of bias (Consolidated Standards of Reporting Trials)	seven studies had unclear risk of bias while two studies had high risk of bias since the outcome assessment was not performed blindly.	Yes	For meta-analysis random-effect models were used in Review Manag-er (RevMan) version 5.0 software. For visual detection and quantitative analysis of the publication bias in each result, the funnel plot and trim and fill method were used in Additional Statistical Software Package (STATA version 16, STATA Corp., College Station, TX, USA).	VAS	For both air blast and tactile test- Laser therapy was significantly more effective than non-laser treatments. The air blast test with higher sensitivity showed significant superiority of laser therapy immediately and at one week after treatment. At other follow-up times, the results of both modalities were the same. However, the tactile test showed the superiority of laser therapy only at 1 week and 1 month. Long-term results were not significant for both air-blast and tactile test.	For the air blast test- I^2^ = 85% heterogeneity. Not mentioned for tactile test.	Follow-up period- Immediate to 18 months. Nd: YAG lasers with no limitation in power or management method. Duration of air blast test- ranging from 1 s- up to 30 s (not mentioned in one study) Limitations- small sample size (both in number of studies and number of patients evaluated in each study), only 2 studies with long-term follow-ups, and inability to assess the efficiency of different desensitizing agents separately.
Bellal et al/2021 [27]	Risk of bias - RoB 1 (The Cochrane Collaboration tool for RCTs (version 5.4, Review Manager (RevMan)	only 1 study showed high risk, 4 studies showed moderate risk, and 1 study showed low risk	Yes	Random effect model was employed as the studies were not functionally the same. Forest plots were used to graphically represent the meta-analysis outcome. Funnel plots were used to evaluate the possibility of publication bias.	VAS	Both immediate and one-month post effect- Laser treatment led to a reduction in VAS scores across all controlled studies except a study conducted by Lier et al. Immediate and 1 month efficacy was statistically significant.	Immediate effect: I^2^ = 96%; One month post treatment I^2^ = 97%	Follow-up period- Immediate, 5 min, 1/4/16 weeks, 1/2/3/6 months. Adverse effects included-none found. All included studies showed a range of laser parameters and techniques/points of application. Small number of studies and narrow field of research (near infrared lasers)
Carneiro et al/2022 [28]	Risk of bias - RoB 1 (The Cochrane Handbook for Systematic Reviews of Interventions 5.1.0)	most studies were classified as low risk of bias. One of the articles could not be classified due to the lack of information and the unsuccessful attempt to contact the authors by email, and another study was classified as high risk	No due to high heterogeneity (I^2^ = 87%)	NA	VAS, MVAS, VRS	The studies reviewed indicated that LLLT showed promise in preventing tooth sensitivity after bleaching. The majority of the studies assessed (five) indicated that LLLT had reduced or even prevented tooth sensitivity. On the other hand, one study did not indicate efficient outcomes.	I^2^ = 87%	Follow-up periods- one performed the assessment for 28 days, others (two) for 21 days, 15 days and 7 days, and another for up to 48 h after each bleaching session, which totalled two. No adverse effects reported
Cerqueira et al/2025 [40]	Risk of bias - RoB 2	only one study was assessed as having “some concerns”, rest have low risk	No	NA	VAS, VAS + 14-item Oral Health Impact Profile (OHIP-14)	The combination with LLT and desensitizing agent in MIH treatment can reduce long-term sensitivity. Moreover, the application of LLT alone exerts an immediate effect on teeth affected by MIH. Combining laser therapy with a varnish resulted in significant differences, with a reduction of 93% in sensitivity (one study).	NA	Follow-up periods- Immediate, 48 h, 1/2/3/4 weeks, 1/3/6/9/12/18/24 months. Reported the lack of a standardized protocol for the number of sessions or the frequency between them and no consensus in the studies regarding irradiation time. In addition to DH treatment, two studies aimed to decontaminate cavities caused by caries lesions and microbiologically evaluated the presence of lactobacilli and streptococci after removing carious tissue with a curette. Regarding the location of laser irradiation, the studies lacked a uniform approach, with some studies applying the laser directly to the affected cavity. This heterogeneity may have occurred because the MIH treatment must be individualized for each patient.
Chen et al/2025 [36]	RoB (The Cochrane Collaboration’s tool for assessing the risk of bias in randomized trials), GRADE	Four studies showed a high risk of bias, seventeen showed a moderate risk of bias, and four showed a low risk of bias. GRADE- Very Low quality of evidence	Yes	random effect model	VAS, NRS, face scale, or Uchida scale	The long-term air blast groups showed significant differences in lasers compared to topical desensitizing agents, while others showed no significant difference. Sensitivity analysis showed that the medium-term air blast group and the short- and medium-term tactile groups lacked robustness	I2 = 62.21–98.63%	Adverse events reported by two studies- one reported-Laser, five reports of mild discomfort, and two reports of temporary tooth sensitivity. Agents, eight reports of mild discomfort, and five of temporary tooth sensitivity. The other one reported-During laser irradiation, all the patients reported pain There were no limits on laser type, power, frequency, intervention period, or management method. There were no limits on the type and concentration of topical desensitizing agents, intervention period, or management method. *15/25 reported no. of patients **18/25 reported no. of teeth one study used " jaw quadrant” as a unit.
He et al/2011 [29]	Risk of bias randomised controlled trials - RoB 1 (the Cochrane Handbook for Systematic Reviews of Interventions)	One trial was classified as A level evidence (high quality and low risk of bias), five trials were rated as B level evidence (moderate quality and risk of bias), and two trials were classified as C level evidence (low quality and high risk of bias).	No due to high heterogeneity	NA	VAS, VRS, a scale of 0–5 points (tactile stimulation), Uchida scale, Evaporative scale, Arbitrary pain scale in 4 degrees	The results obtained from the eight retrieved studies were conflicting. A major part of them showed positive outcomes. The systematic review of the literature indicates a trend toward a slight clinical advantage following laser treatment of dentinal hypersensitivity compared to application of topical medicaments. Six studies reported significant reduction in the DH after laser treatment compared to control group. Two studies reported no significant difference.	NA	Follow-up period - up to 6 months. High heterogeneity among analyzed studies (lack of RCTs, small sample size, different types of lasers and follow-up periods, different ways to evaluate outcomes, inadequate control of confounding factors associated with DH) No adverse effects reported.
Hu et al/2019 [31]	Risk of Bias - RoB 1 (the Cochrane collaboration tool), quality of evidence (GRADE tool)	Only 1 study had a low risk, 13 had a moderate risk, and the remaining 8 had a high risk Lasers compared with placebo or no treatment had moderate-quality immediate and long-term effects on DH	Yes	Funnel plots were used to evaluate the possibility of publication bias.	VAS, NRS	The results showed that all types of lasers had better immediate and long-term desensitizing effects on DH than negative controls.	I^2^ = 90% − 98%	Follow-up period - immediately after treatment to 6 months Types of lasers used-Er, Cr: YSGG, erbium, chromium: yttrium-scandium-gallium garnet; Er: YAG, erbium-doped yttrium aluminium garnet; GaAlAs, gallium-aluminum-arsenide; Nd: YAG, neodymium doped yttrium aluminium garnet. Lasers- no limits on power, frequency, intervention period, or management method
Kong et al/2019 [32]	Risk of Bias - RoB 1 (The Cochrane Collaboration tool), quality of evidence (GRADE tool), sensitivity analysis and inconsistency assessment	only one study had a low risk, six had a moderate risk, and the other four had a high risk The quality of evidence of the final included studies indicated lasers vs. placebo or no treatment with a moderate or low quality in the immediate and long term	Network meta-analysis	Funnel plots were used to evaluate the possibility of publication bias, random effects model for meta-analysis and random effects network within a Bayesian framework model using Markov chain Monte Carlo methods in ADDIS 1.16	VAS	All four types of lasers had a better desensitizing effect than controls immediately after treatment and over the long term, but there were no significant differences among the four different lasers. There was a significant placebo effect immediately after treatment. The laser with the highest probability of being the most effective treatment for DH was Er, Cr: YSGG immediately after treatment and over the long term (73% and 47%, respectively). Inconsistency assessment showed no significant inconsistency between comparisons except Er: YAG versus Nd: YAG (*P* < 0.05), indicating that it conforms to the similarity assumption.	Mentioned in Methodology but not reported in Results.	Follow-up period- immediately after treatment to 6 months. The cumulative probabilities of being the most efficacious treatments were 73% for Er, Cr: YSGG followed by Nd: YAG, GaAlAs, Er: YAG, placebo, and no treatment, sequentially. Based on the network of long-term (1-month) desensitization effects, the cumulative probabilities of being the most efficacious treatments were 47% for Er, Cr: YSGG followed by Nd: YAG, Er: YAG, GaAlAs, placebo, and no treatment, sequentially. There were no limits on the power or management method, but the first follow-up time had to be less than 30 min and the long-term follow-up time was 1 month Sample sizes of the included studies were insufficient.
Lestari et al/2023 [39]	Quality assessment (JBI’s Checklist for Randomized Controlled Trials)	NA	No	NA	VAS, VRS, Yeaple score, LDF	All literature presented a significant reduction in dentin hypersensitivity at follow-up immediately after the intervention up to 3 to 6 months after treatment. One study stated that the treatment of DH using NaF only started to show a significant reduction in the pain score (VAS) from the first week of follow-up. However, the use of glutaraldehyde as desensitizing agents showed better performance than the Nd: YAG laser in the reduction of dentin hypersensitivity (Yeaple score assessment) and suggested the use of the Er, Cr: YSGG laser combined with glutaraldehyde is more effective in treating DH. Nd: YAG presents a significant immediate reduction of DH, but uncertain long-term effects.	NA	Reported one study (Ozlem K et al.) twice in Table 1. All studies had more than one follow-up period, but most studies performed short-term (up to 1 day) and medium-term (2–7 days, up to 30 days), while long-term evaluation (up to 6 months) has only been performed in 3 out of 6 studies. Limitation- short follow-up period.
Lin et al/2013 [33]	NA	NA	Network meta-analysis, Meta-regressions with covariates, Standard pair-wise meta-analyses	For network meta-analysis- the Bayesian hierarchical random-effects modelling. The standard pair-wise random- effects meta-analysis, heterogeneity between studies and meta regressions were performed using statistical software STATA (version 11.2, StataCorp LP, College Station, TX, USA).	VAS, VRS	Most active treatment options including physical occlusion group, chemical occlusion group, laser therapy group and combined treatment group had significantly better treatment outcome than placebo group. However, nerve desensitization, did not show a significant benefit as reported by network meta-analysis. Comparisons of the five active treatment groups showed no significant differences. The study designs including split-mouth-designed trials versus parallel-designed trials, follow-up periods, and multiple treatment courses versus single treatment showed no significant differences. Meta-regressions with covariates including the length of follow-ups, study design, multiple treatment courses and interaction terms among them were tested and none of the results was significant (*p* > 0.05).	The statistical heterogeneity of these comparisons was high (I^2^ = 88.6% ~96.7%) except the comparison between physical occlusion group and combined treatment group (I^2^ = 0.0%, *p* = 0.71).	Literature was searched uptill Dec 2011, and one study included from 2012. Follow-up period - immediate to 6–9 months. No adverse events reported. Exact number of participants and teeth could not be calculated
Machado et al/2018 [9]	NA	NA	No due to high heterogeneity	NA	VAS, numerical scales	Reported positive effects for low-power laser irradiation. Also reported that application of other desensitizer agents was effective, as shown in the placebo group of the studied conducted by Vieira et al. None of the studies affirmed that the laser equipment could show higher costs than those of the other in-office treatments. Reported statistically significant differences, also presenting a relative positive result in terms of decreasing pain	NA	Follow-up period - immediate to 6 months. No adverse effects reported. High heterogeneity in terms of the different types of lasers, wavelengths, energy settings, number of sessions, and variety of follow-up periods used. Recommended oral care, dietary instructions and occlusal adjustment, if needed, before treatment planning.
Mahdian et al/2021 [1]	Risk of Bias - RoB 1; Quality of Evidence (GRADE tool)	Five studies at overall low risk of bias, 13 at unclear, and five at high risk of bias. Allocation concealment, blinding and randomization and attrition bias received highest % of unclear risk of bias. Overall certainty of the evidence for all types of lasers was low to very low	Yes	Fixed-effect and random-effect models, generic inverse variance method for split-mouth trials	VAS, Air-sensitivity scale (0 to 3)	Lasers vs. placebo or no treatment may reduce pain intensity when tested through air blast stimuli at short-term (MD −2.24, 95% CI −3.55 to −0.93; *P* = 0.0008; 13 studies, 978 teeth; low-certainty evidence), medium term (MD −2.46, 95% CI −3.57 to −1.35; *P* < 0.0001; 11 studies, 1007 teeth; very low-certainty evidence), and long-term (MD −2.60, 95% CI −4.47 to −0.73; *P* = 0.006; 5 studies, 564 teeth; very low-certainty evidence). Similarly, lasers vs. placebo or no treatment may reduce pain intensity when tested through when tested through tactile stimuli at short-term (MD −0.67, 95% CI −1.31 to −0.03; *P* = 0.04; 8 studies, 506 teeth; low-certainty evidence) and medium-term (MD −1.73, 95% CI −3.17 to −0.30; *P* = 0.02; 9 studies, 591 teeth; very low-certainty evidence), but there was insufficient evidence in the long-term (MD −3.52, 95% CI −10.37 to 3.33; *P* = 0.31; 2 studies, 184 teeth; very low-certainty evidence). There was no significant difference for the comparison between Er, Cr: YSSG laser and placebo. Quantitative analysis of all types of lasers combined, revealed a statistically significant reduction in DH in short-, medium-, and long-term compared to placebo/no treatment.	NA	No adverse events reported. No studies investigated the impact of laser treatment on participants’ quality of life. No studies carried out intention-to-treat analysis. High heterogeneity among included studies.
Marto et al/2019 [2]	Risk of Bias - RoB 1 (Cochrane Handbook of Systematic Reviews of Interventions)	most studies present randomisation and allocation concealment and are considered as having low risk of selection bias. (Blinding intervention of participants and personnel in clinical procedures was impossible in several studies (32.4%) or was not explained in the description of the clinical study (8.1%). This performance bias was more common since the characteristics of products or techniques were easy to distinguish from each other. Blinding evaluation of the results was possible in most studies, with minimal risk of bias (74.3%). Attrition bias with incomplete outcome data, and reporting bias were present in 10.8% of RCT studies, which had to be excluded from the quantitative analysis. Other biases were of minimal risk in all scrutinised studies.)	No due to high heterogeneity	NA	VAS, Evaporative stimulation method	Lasers, glutaraldehyde, and glass ionomer cements induced statistically significant differences relative to placebo at different follow-up times. No significant differences within a specific group over time. Other treatments demonstrated reduced DH relative to placebo with statistical significance in medium‐term follow‐up. Hydroxyapatite treatment resulted in a statistically significant reduction in DH relative to placebo from 8 to 15 days and increasing significance over time. Potassium nitrate treatment revealed statistically significant differences in the reduction in DH only in the follow‐up of 1‐3 months. In laser treatments, there were no statistically significant differences over time in any follow‐up between high and low power laser	NA	Follow- up periods − 1 day, from 2 to 7 days, from 8 to 15 days, from 15 to 30 days, from 1 to 6 months and more than 6 months. High heterogeneity in the laser group mentioned in the discussion. Limitations - short follow‐up, small sample in some groups.
Mohammadian et al/2025 [43]	RoB 2	All three studies clearly described randomization methods; however, allocation concealment was not adequately detailed, resulting in an unclear risk. Deviations from planned interventions were low. The risk of bias due to missing data was unclear due to insufficient reporting in two studies. Bias in outcome measurement was unclear in studies since none of them mentioned whether the outcome assessors were blinded to the intervention groups. The risk of selective reporting was low in all studies.	NA	NA	VAS	Intragroup changes- All studies demonstrated significant reductions in DH within each group. Intergroup changes- Combination therapy consistently showed the greatest reduction in DH. diode laser therapy, particularly when combined with 5% NaF varnish, may offer superior and longer-lasting relief from DH compared to either modality alone.	NA	No adverse events reported. Follow up- Immediately, 24 h, 1/2 weeks, 1/2/3/6 months One study has 7 dropouts
Oliveira da Rosa et al/2013 [30]	NA	NA	No due to high heterogeneity	NA	VAS	No therapy could be considered ideal. Cervitec Plus, SE Bond & Protect Liner F, Laser, and Iontophoresis have shown satisfactory posttreatment results between 3 and 6 months. In one study, in a 4-month posttreatment evaluation, the effects of activated Nd: YAG laser were not statistically significant or different from the placebo group (non-activated laser) in reducing pain.	NA	Follow-up period − 3 months. Lack of clinical trials evaluating dentin desensitizing agents over 6 months. High heterogeneity of study design, material tested, no. of treatment sessions and variety of follow-up periods. Reason reported behind decreased posttreatment efficacy- wash-out of desensitizing agents over time 9 studies evaluated laser therapy out of 17.
Pion et al/2023 [4]	Risk of Bias - RoB 1 (Cochrane Handbook for the Development of Systematic Intervention Reviews)	Many studies had unclear risk of bias due to missing information. Only 40% detailed random sequence generation, and just 4 described allocation concealment. Among low-risk studies, 68% and 65% had proper blinding of participants/personnel and outcome assessments, respectively. Most studies had acceptable attrition rates (< 20%). However, 34% had a high risk of other biases, such as inconsistent laser application or use of fluoride-free toothpaste. In the meta-analyzed studies, most (except for one) reported randomization, but two lacked allocation concealment details and two had high risk from other biases. Two single-arm studies were excluded from bias assessment.	meta-analysis of random effects, network meta-analysis, Bayesian analysis of mixed treatment comparisons (MTC)	MTC analyses were computed using both fixed and random models, and the merit of fit for the models was quantified using the residual deviation and the residue information criterion (RIC). A node split analysis for unreliability was performed for the pairs with both direct and indirect evidence. Meta-analyses and network meta-analyses were performed using the R package (GeMTC), v. 3.6.1 (R Foundation for Statistical Computing, Vienna, Austria).	VAS, number scale, Ushida scale, pain scale (3/4 degrees)	Statistically significant differences found between the average pain before and after 3 months of treatment with high- and low-power lasers. High-power laser showed a greater tendency to reduce the pain levels after 3 months of treatment as compared to the low-power laser, but without a statistically significant difference. Most of the protocols using lasers with or without another desensitizing agent reduced the pain levels during the follow-up period. Two studies with 18 months follow-up reported significantly decreased pain for all treatments.	I^2^ = 89% (high power) and 91% (low power)	Follow-up periods- most frequently 6 months (55%), two studies 18 months.
Rezazadeh et al/2019 [34]	NA	NA	NA	NA	VAS, VRS, NRS, VAS + plaque index, ABS, (0–4-degree scale) (Uchida criteria)	Nd-YAG (neodymium-doped yttrium aluminium garnet) and diode lasers reduced DH after bleaching. Lasers showed efficiency in prevention or management of DH associated with cervical restorations and relief of DH following scaling and root planning. A few studies disputed the benefits of lasers compared to placebo. Among various types of lasers, the application of Nd: YAG laser has shown the best results in DH treatment. Some studies have reported no significant difference between laser and other desensitizing agents, and most of the studies proposed better results (both rapid and long lasting) in combined modalities.	NA	Follow up period - Immediate to 6 months
Sgolastra et al/2011 [35]	Risk of Bias - RoB 1 and Quality Assessment (CONSORT statement for the evaluation of randomized clinical trials, Cochrane Handbook for Systematic Reviews of Interventions)	The risk of bias before contact with the authors was estimated to be high for all 3 studies (inter-reviewer agreement, kappa = 1.0). After contact with the authors, the risk of bias was considered low for the study of Lier et al. and high for the studies of Birang et al. and Vieira et al. (inter-reviewer agreement, kappa = 1.0)	No due to high heterogeneity	NA	VAS scale	Laser treatment resulted in not significant reduction of DH compared with placebo laser treatment. In one study, VAS was significantly reduced compared with baseline at all time points in all groups. In other two studies, a significant decrease in VAS was observed in both groups at each time point, but no significant difference was observed between groups throughout the study period.	NA	No pulp damages or major adverse effects were reported. High heterogeneity in terms of the different laser types, wavelengths, energy settings, number of treatment sessions, and variety of follow-up periods. Small sample size (56 patients across three studies).
Sgolastra et al/2013 [42]	Risk of Bias - RoB 1 and Quality Assessment (revised recommendation of the CONSORT statement)	Three studies (Sicilia et al., 2009; Yilmaz et al., 2011a, b) were at low risk of bias; all of the remaining studies were considered to be at high risk of bias. The most frequent unsatisfactory criteria were the lack of a sample size calculation and inadequate or unreported methods of randomization and allocation concealment.	Yes	Random-effects model (Der Simonian and Laird model), Forest plots for each meta-analysis	VAS, VRS	Er: YAG, Nd: YAG, and GaAlAs lasers showed significant reduction in pain level between baseline and follow-up. For Er, Cr: YSSG vs. placebo, the meta-analysis result from 3 studies demonstrated a non-significant change.	I^2^ = 97–99%	Follow-up periods - immediately to 6 months. No cost-effectiveness analysis could be performed because none of the included studies reported any information on this issue. No adverse effects reported, only one study reported pain immediately after treatment in two patients. High and significant heterogeneity in all 4 comparisons which could be contributed to differences in the study design (parallel vs. split-mouth), laser parameters/settings, methods of DH assessment, types of stimulation, and follow-up times.
Shakeel et al/2022 [38]	NA	NA	NA	NA	Evaporative stimulus, Uchida scale	Five studies reported reduced sensitivity with GaAlAs lasers. Greater efficiency was reported when combined laser with desensitizing agents compared to laser treatment alone. One study reported no significant differences due to the similarity between laser characteristics.	NA	The Cochrane risk assessment tool is mentioned in the abstract but not in the main text. No risk assessment results are presented. Specific focus on GaAIAs was not justified.
West et al/2015 [41]	Risk of Bias - RoB 1 and Quality Assessment	Strontium/Stannous fluoride/PVA/MA – Polymers- moderate risk CSPS-5 studies have high risk, remaining-moderate risk, quality of evidence-low Oxalates/Resin based materials/Arginine and calcium carbonate (A/C)- one study has high risk, remaining have moderate risk Varnishes/Lasers/Potassium- two are high risk, remaining are moderate risk ACP-CPP- One study has low risk, one has moderate and one has high risk of bias	No due to high heterogeneity	NA	VAS, Schiff, Yeaple, air blast	Of the eight studies, seven compared efficacies with a variety of comparator products. Only Gerschman et al. (1994) used a negative control. Laser treatment consistently decreased the pain associated with DH. However, studies demonstrated minimal superiority over comparator agents, insufficient evidence to recommend laser treatment compared to other professionally applied agents.	NA	Follow-up periods- immediate to 25.2 months Heterogeneity present between studies * 21/105 mentioned no. of teeth
Zhou et al/2021 [37]	Risk of Bias and Quality Assessment (the Cochrane Risk of Bias tool)	four, six and three studies showed low, moderate and high risks of bias, respectively. Low quality of evidence.	Yes	Random effect model	VAS	All comparisons, except for the 3-month Nd: YAG laser parallel group and 6-month diode laser group, showed that the clinical efficacy of laser for DH was not significantly different from topical desensitizing agents. Low-quality evidence found which was insufficient to draw any conclusions regarding the superiority of lasers or conventional topical desensitizing agents in the treatment of DH. The combined results at each follow-up indicated no significant difference between Nd: YAG laser and topical desensitizing agent treatment.	Immediate effect-I^2^ = 84%, *p* < 0.0001 After 1 week- (I^2^ = 72%, *p* = 0.007) After 1 month- I^2^ = 90%, *p* < 0.00001 After 3 months- (I^2^ = 94%, *p* < 0.00001 After 6 months- (I^2^ = 0%, *p* = 0.91)	Follow-up periods - immediate to 9 months. No adverse effects reported. Given the low cost and ease of use of topical desensitizing agents, clinicians are recommended to use them as a routine treatment for DH. On the other hand, lasers can be used in cases where topical desensitizing agents are ineffective, taking into account the multifactorial and multi-aetiological nature of DH. Types of lasers- Nd: YAG, GaAIAs, Diode lasers Study highlights that comprehensive management, such as oral hygiene instruction, avoidance of excessive acidic diet and incorrect toothbrushing habits, and improvement of incorrect occlusal pattern caused by wear or periodontal disease, should be included in patients with DH, irrespective of the desensitisation methods used. High heterogeneity in most comparisons in this study; the subgroup analysis alleviates heterogeneity but cannot eliminate it, indicating that the difference in study design (parallel vs. split-mouth) is not the sole source of heterogeneity.
*VAS* visual analogue scale, *VRS* verbal rating scale, *LDF* laser doppler flowmetry, *NRS* numerical rating scale, *ABS* air blast scale, *NA* not available								

### Overlap of primary studies

Overlap analysis included 206 primary studies across 25 systematic reviews (Appendix E)**.** The CA calculation based on all included primary studies (including double counting) across the citation matrix (number of rows multiplied by the number of columns) showed a score of 7.8 indicating a moderate level of duplication. The corrected measure, CCA, interpreted as the area covered after eliminating the inclusion of all primary publications the first time they are counted, resulted in a score of 3.9, indicating a slight degree overlap of primary studies:

$$\:CA=\frac{N}{rc}=\:\frac{402}{206\times25}=0.078=7.8\%\:$$

$$\:CA=\frac{(N-r)}{(rc-r)}\:=\:\frac{(402-206)}{(206\times25-206)}=0.039=3.9\%$$

### Quality assessment

Seven reviews were rated as high quality [1, 7, 10, 27, 31, 32, 36], 10 as moderate quality [2–4, 8, 9, 28, 29, 37, 42, 43], and 8 as low quality **(**Table 4**)** [30, 33–35, 38–41]. Only 3 out of 25 reviews adequately addressed all JBI Critical Appraisal checklist items [1, 32, 36]. On the other end of the spectrum, 1 review did not adequately address any of the items from the JBI Critical Appraisal checklist [38]. Looking at specific items, the one that was mostly adequately addressed was “Is the review question clearly and explicitly stated?”, with only 2 unclear ratings [30, 38]. “Was the likelihood of publication bias assessed?” was not addressed in the majority of reviews [2–4, 9, 28–30, 33–35, 38, 41–43]. Other items of concern are “Was critical appraisal conducted by two or more reviewers independently?”, “Were the criteria for appraising studies appropriate?”, “Were the methods used to combine studies appropriate?”, and “Were the specific directives for new research appropriate?” Table 5 summarizes the overall quality of the included reviews and its impact on the conclusions of this umbrella review. While laser therapy generally showed positive effects on DH, the limited quality and methodological weaknesses of a number of included reviews affected the certainty of the conclusions. This table highlights how evidence quality informed our cautious interpretation and conclusions.

**Table 4:**
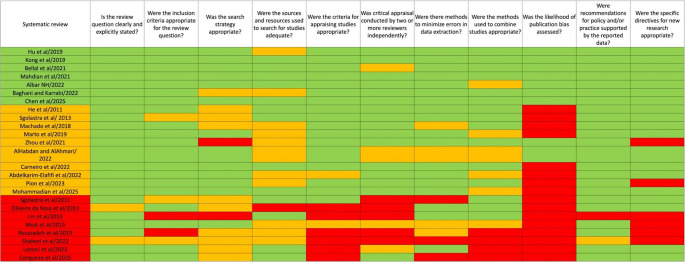
JBI critical appraisal checklist items

Colour coding for each item: green – “yes”; orange – “unclear”; red – “No”. Colour coding for overall quality rating of reviews: green – “high quality”; orange – “moderate quality”; red – “low quality”

**Table 5:** Summary of evidence quality and its influence on review conclusions

Author/Year	JBI quality rating	Main findings	Impact on conclusions
Abdelkarim-Elafifi et al/2022 [3]	Moderate	Lasers consistently reduced pain across studies	Weighted as supportive, but heterogeneity noted
Albar (2022) [7]	High	Lasers effective, but GLUMA superior for immediate effect; lasers better for long term	Weighted for nuanced interpretation (agent vs. laser)
AlHabdan and AlAhmari/2022 [8]	Moderate	Er, Cr: YSGG effective	Weighted as supportive, but heterogeneity noted
Baghani & Karrabi (2022) [10]	High	Laser therapy significantly better than controls in air blast and tactile tests at early follow-ups	Moderate weight; supports short-term benefits, limited long-term evidence
Bellal et al. (2021) [27]	High	Laser treatment reduced DH at baseline and 1 month; heterogeneity very high	Weighted but cautiously interpreted due to heterogeneity
Carneiro et al/2022 [28]	Moderate	LLLT reduced/prevented sensitivity in most studies	Moderately weighted; added support but increased heterogeneity
Cerqueira et al/2025 [40]	Low	LLLT alone gave immediate relief in MIH; combined with desensitizer/varnish, markedly reduced long-term sensitivity	Minimal impact due to low quality and inconsistency. Weak methodology
Chen et al. (2025) [36]	High	Long-term air-blast favoured lasers; other results mixed, low robustness	Strongly weighted; supported use over conventional agents
He et al/2011 [29]	Moderate	Most studies showed lasers reduced DH vs. controls; some reported no difference	Moderately weighted but limited by older evidence base
Hu et al. (2019) [31]	High	All types of lasers superior to placebo for immediate and long-term relief	Strongly weighted; supported consistent efficacy
Kong et al. (2019) [32]	High	All four laser types better than controls; Er, Cr: YSGG had highest probability of efficacy	Strongly weighted; informed comparison across laser types
Lestari et al/2023 [39]	Low	DH reduced up to 3–6 months; NaF effective from week 1; glutaraldehyde ± Er, Cr: YSGG laser most effective.	Minimal impact; downgraded due to low quality evidence
Lin et al/2013 [33]	Low	All active treatments except nerve desensitization better than placebo; no differences among active treatments or study factors	Minimal impact; weak support for laser therapy
Machado et al. (2018) [9]	Moderate	Positive effects of low-power lasers but heterogeneity high	Cautiously weighted
Mahdian et al. (2021) [1]	High	Lasers vs. placebo/no treatment showed significant short- and medium-term reductions in DH; evidence certainty low–very low	Strongly weighted; key support for short-term efficacy. But cautiously interpreted due to heterogeneity
Marto et al. (2019) [2]	Moderate	Lasers and other desensitizers superior to placebo; no consistent differences within laser groups	Moderately weighted; evidence added heterogeneity
Mohammadian et al/2025 [43]	Moderate	Intragroup: significant DH reduction; Intergroup: combination therapy, especially diode laser + 5% NaF, most effective and longer-lasting.	Moderately weighted; useful but not definitive
Oliveira da Rosa et al. (2013) [30]	Low	Mixed results, some showing no significant effect vs. placebo	Minimal impact
Pion et al/2023 [4]	Moderate	All lasers reduced pain; high-power slightly better at 3 months; 18-month follow-up showed significant reduction for all	Moderately weighted; added supportive evidence
Rezazadeh et al. (2019) [34]	Low	Some evidence for Nd: YAG, but mixed with contradictory results	Minimal impact; not emphasized due to low quality
Sgolastra et al. (2011) [35]	Low	Lasers not significantly better than placebo; small sample	Minimal impact; low confidence in findings
Sgolastra et al/2013 [42]	Moderate	Er: YAG, Nd: YAG, GaAlAs lasers reduced pain; Er, Cr: YSGG vs. placebo showed non-significant change (3-study meta-analysis).	Moderately weighted but interpreted with caution
Shakeel et al/2022 [38]	Low	GaAlAs lasers effective; combination with desensitizing agents superior; one study showed no difference	Minimal impact due to poor methodological strength
West et al/2015 [41]	Low	Lasers reduced DH pain but showed minimal advantage over comparators; insufficient evidence to prefer lasers over other professional treatments.	Minimal impact; downgraded due to weak evidence
Zhou et al/2021 [37]	Moderate	Lasers generally comparable to topical agents; low-quality evidence; no significant difference for Nd: YAG	Moderately weighted; contributed to pooled effect

### Risk of bias assessment

RoB assessment is presented in Table 6. Only 2 systematic reviews [1, 36] were assigned low RoB, whilst all other included reviews were assigned high RoB. Domains 1 and 4 were most common sources of high RoB with 19 “no” answers each for various signalling questions in these domains. All 4 domains were rated as high RoB in 8 reviews [3, 9, 30, 33–35, 38, 39].

Appendix F contains additional comments pertaining to specific signalling questions that justify the rating. Common concerns were related to the lack of protocol or registration; discrepancies in comparators/interventions/outcomes (Domain 1); incomplete search strategy, limited databases, no grey literature or manual searches (Domain 2); unclear number of reviewers or single reviewer only, RoB assessment missing or unreported (Domain 3) and lack of heterogeneity assessment, funnel plot or sensitivity analysis, overstated conclusions (Domain 4).

**Table 6:**
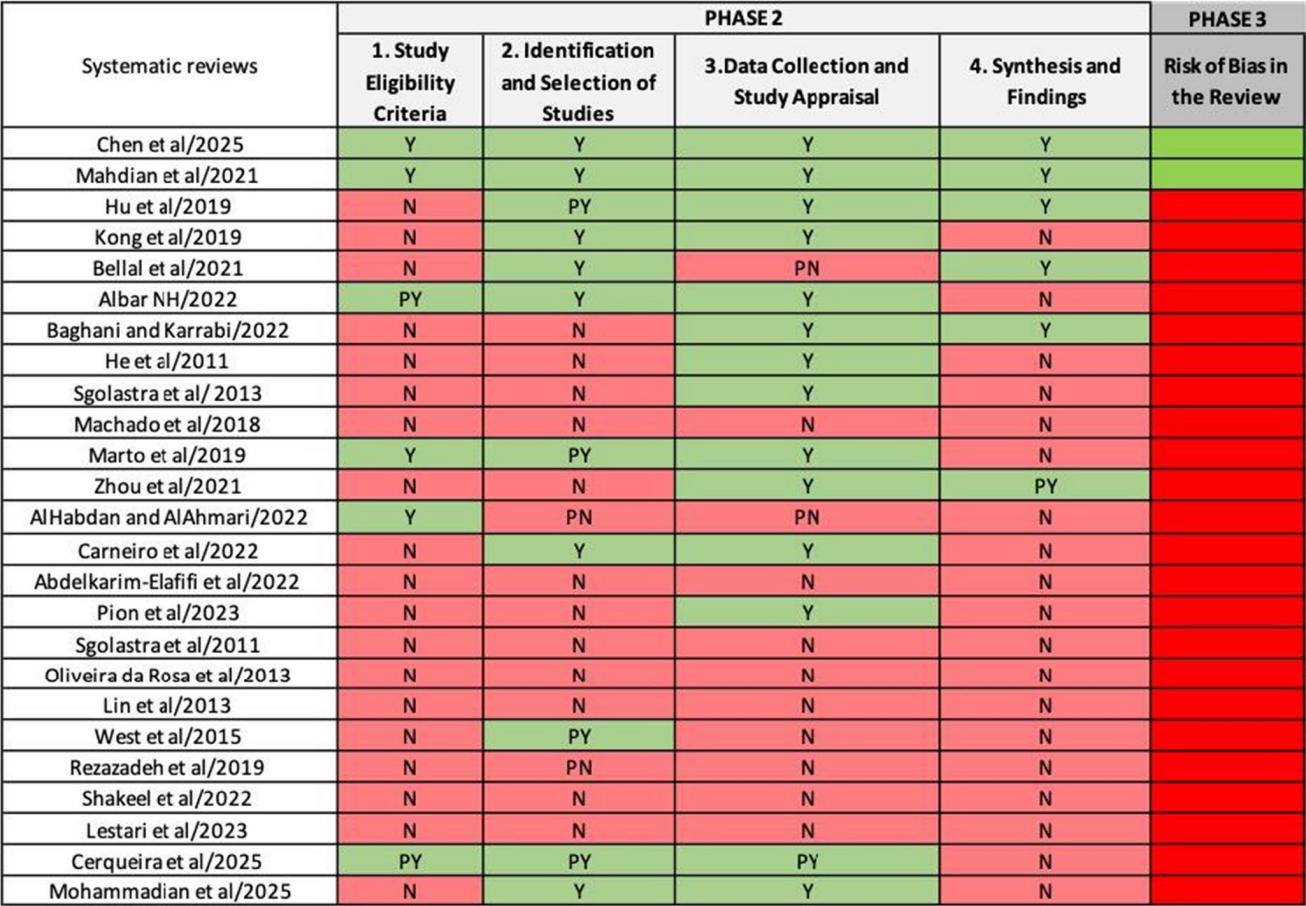
Risk of bias assessment

Colour coding for each item: green – “yes (Y)” or” probably yes (PY)”; red – “No (N)” or” probably no (PN)”. Colour coding for overall risk of bias: green – “low risk of bias”; red – “high risk of bias”. No reviews were rated as “unclear risk of bias”

### Synthesis of findings

Main findings indicated improvement in the clinical presentation, with reduced pain either from laser treatment alone [1–4, 7–10, 27, 29, 31–33, 37–39, 41, 42] or in combination with other desensitizing agents [28, 33, 40]. Low-level laser treatment helped alleviate or even prevent pain in the majority of primary studies after bleaching [28]. The same pain-reducing or pain-preventing effect after bleaching was reported for Nd: YAG laser [34]. Low-level laser treatment alone reduced pain in patients with MIH immediately, whilst a long-term positive effect was reported when laser was combined with a varnish [40].

Detailed stratification of results by laser type, comparator, or follow-up is hindered by the variability of findings and differences in evidence quality of the included reviews. Some generalised stratified findings indicate that a combined laser treatment with another desensitizing agent performs better in terms of pain management compared to laser alone [28, 40]. As for comparison against other treatment modalities, laser treatment usually provided greater pain relief than no treatment [31, 32] or placebo treatment [1, 3, 31, 32, 34], but there were some conflicting findings of no difference [30, 35, 42]. These findings were underscored by 4 high-quality reviews that reported greater reduction in pain for lasers compared to placebo or no treatment in short, medium, and long term [1, 27, 31, 32], despite the reported evidence being of low certainty [1]. Comparing specific types of lasers, no differences were found between low- and high-power laser treatment [2, 4, 7] or various laser types, specifically GaAlAs, Nd: YAG, Er: YAG, Er, Cr: YSGG, CO₂ [32, 42].

Several studies found no significant effects comparing laser to other forms of treatment [2, 9, 29, 32–34, 37, 41]. The use of glutaraldehyde as a desensitizing agent showed better performance than the Nd: YAG laser in the reduction of dentin hypersensitivity (Yeaple score assessment) and suggested the use of the Er, Cr: YSGG laser combined with glutaraldehyde was more effective in treating DH [39]. A similar finding was reported for Gluma desensitizing agent in the immediate post-treatment period, with laser providing a longer lasting effect [7]. Laser treatment was found to be more effective than control desensitizing agents in the short-term, up to 1 month [10].

In terms of follow-up, several reviews reported consistent short-term reductions in DH following laser therapy, and 8 of 25 studies provided long-term follow-up data (beyond 6 months), demonstrating sustained benefits [2–4, 10, 33, 37, 40, 41].

## Discussion

This umbrella review provides a comprehensive synthesis of 25 systematic reviews evaluating the effectiveness of laser therapy in managing DH. By consolidating a wide range of laser interventions, comparator agents, outcome measures, and patient populations, we offer a high-level overview of the current evidence landscape.

To our knowledge, there is only one other review to examine both the clinical finding, the methodological quality of systematic reviews in this area and citation overlap in primary studies to evaluate redundancy [20]. The results of our umbrella review support earlier findings that laser therapy can reduce DH. However, our expanded scope provides additional insights. By incorporating evidence from 25 systematic reviews and including clinical trials and cohort studies, we were able to capture a more comprehensive and up-to-date evidence base than the earlier umbrella review, which was restricted to nine systematic reviews published before November 2022. Moreover, our inclusion of comparisons with other desensitizing agents highlights that, while lasers consistently outperform placebo, their relative effectiveness against alternative treatments remains less certain. Importantly, by examining secondary outcomes such as recurrence, cost-effectiveness, and safety, our findings address practical considerations that were not explored previously, thereby enhancing the clinical relevance of this evidence.

More than half of the analysed reviews were published in the last 5 years, reflecting the ongoing high prevalence of DH [5]. This finding indicates increased awareness of the need to treat DH but also a lack of standardized treatment approach. It also reflects a growing clinical and research interest in the application of laser therapy for DH. The need for an umbrella review is underscored by an expanding volume of systematic reviews in recent years which may lead to redundancy, conflicting conclusions, and challenges for clinicians seeking to interpret the evidence base.

### Methodological limitations of existing evidence

The most striking finding of this umbrella review is the heterogeneity in investigative approach. A variety of treatment options are understandable due to the lack of a gold standard approach. However, differences in summarizing the evidence and, in particular, non-adherence to the recommended standards in reviewing resulted in a number of low-quality and high RoB publications, precluding meaningful clinical recommendations.

Following the JBI Critical Appraisal Checklist for Systematic Reviews and Research Syntheses [21], only 7 reviews were rated as high quality. A number of questions were not adequately addressed, with the likelihood of publication bias being the most problematic. As per JBI guidance, acceptable methods of assessing publication bias are funnel plot analysis, Egger’s or Begg’s test or other regression-based methods to assess funnel plot asymmetry, and discussion of grey literature. Funnel analysis was used in several reviews with meta-analysis, but very little or no attempt was made to address the publication bias in systematic reviews with no additional meta-analytical statistics. This may be due to the lack of understanding of publication bias and its importance or the lack of guidance if specific statistical tests are not employed. In systematic reviews without meta-analysis, alternatives include trial registries, unpublished studies, or grey literature [21]. In the context of laser in DH treatment, publication bias may occur when studies with positive results—confirming the effectiveness of lasers, at least as good as other treatment options—are more likely to be published than studies with negative results, thereby affecting the validity of a systematic review.

While JBI provides a broad view of methodological rigor, RoB assessment using the ROBIS tool allows a more stringent analysis of bias that might affect outcomes and conclusions. This is evident in the architecture of each domain, particularly the extent to which signalling questions probe relevant domains. Methodological concerns identified through the JBI tool were further underscored by the ROBIS tool, with only 2 reviews rated as low RoB, while the remainder demonstrated moderate to high RoB. Domains that were often not adequately addressed involved the lack of protocol registration, inadequate search strategies, and inadequate or insufficient reporting on review processes. All these methodological aspects are not inherent and can be easily rectified if more stringent guidelines were adopted and followed by authors and reinforced by journal editors and reviewers.

This umbrella review did not conduct a new meta-analysis, as the unit of analysis was the systematic review, and re-analysis of primary study data is beyond the scope of an umbrella review. Of the 25 included systematic reviews, 10 reported conducting a meta-analysis. However, substantial heterogeneity (I² >60% to >80%) was commonly observed, limiting the generalizability of the pooled estimates. The remaining 15 reviews presented narrative syntheses, often citing variability in laser types, control groups, outcome measures, and follow-up durations as barriers to meta-analysis [2, 3, 7–9, 28–30, 34, 35, 38–41, 43].

Another important source of heterogeneity across the included reviews was the variation in population characteristics and outcome reporting. While most reviews included adult patients or did not specify age limits, only a few focused on specific populations, such as children [1, 2], or conditions, such as patients with MIH [40] or post-bleaching sensitivity [28]. Although VAS was a commonly adopted outcome measurement tool, with or without additional pain assessment tools, outcome reporting inconsistencies were largely related to differences in pain elicitation methods. Furthermore, patient numbers were generally reported in primary studies, but the data on the number of treated teeth were inconsistently provided or missing entirely in some reviews [2, 4, 8, 27–29, 31–37, 39–42].

The majority of included systematic reviews primarily focused on randomized controlled trials (RCTs), which provide higher-level evidence than other clinical trials. However, a few reviews included other study designs, such as prospective clinical trials, single-arm trials, and in vivo studies without control groups [4, 7, 34, 38]. While study design (e.g. split-mouth vs. parallel) may affect the outcomes of individual clinical trials, this umbrella review focuses on synthesizing evidence at the systematic review level. The influence of individual study design could be explored in future meta-analyses. This variation in study design likely contributed to heterogeneity across reviews. All of the above limits the ability to assess the validity and applicability of the reported treatment effects, potentially reducing the overall generalizability of the findings. It also emphasizes the importance of rigorous critical appraisal when synthesizing such diverse evidence.

### Clinical and policy implications

Dental practice is becoming increasingly reliant on technology. A growing applicability of lasers in dental procedures has been predicted with a compound growth annual rate of 5.7% from 2023 to 2032 [44]. It is therefore reasonable to expect that lasers will continue to be used in DH treatment.

For clinicians, the evidence supports the use of laser therapy as a non-invasive, adjunctive option for managing DH, either alone in combination with other desensitizing agents, particularly for patients unresponsive to conventional treatments or undergoing procedures known to cause sensitivity. A relatively consistent finding was that laser treatment was more effective than placebo or no treatment, whilst the findings comparing lasers with other desensitizing agents were inconclusive and, at times, conflicting. Similarly, current evidence suggests no preference for laser type or power. However, given the short-term nature of most reported outcomes and the heterogeneity in protocols, clinicians should exercise caution and apply shared decision-making when integrating laser treatment into practice. Despite a number of analysed studies highlighting sustained benefits beyond 6 months, the number of long-term studies is limited, and their heterogeneity highlights the need for further high-quality research with extended follow-up.

From a policy and guideline development perspective, our findings highlight the need for standardization in clinical trial design, including the use of core outcome sets, consistent pain assessment tools, and longer follow-up periods. Inconsistent reporting and methodological weaknesses limit the ability of professional societies and policymakers to make evidence-based recommendations. Moreover, the cost and accessibility of laser equipment may pose barriers to widespread implementation, especially in low-resource settings. Economic evaluations comparing laser to conventional treatments are currently lacking and should be prioritized.

### Strengths and limitations of this umbrella review

A major strength of this umbrella review lies in its comprehensive and structured synthesis of existing systematic reviews, combined with a critical appraisal using both the JBI checklist and the ROBIS tool. Additionally, our use of citation matrix and overlap analysis (CCA) allowed us to assess redundancy across reviews — an uncommon but valuable step in umbrella reviews. The corrected covered area (CCA) was found to be 3.9%, indicating a slight overlap among primary studies. This suggests that the included systematic reviews drew on largely distinct sets of primary studies, thereby reducing redundancy and supporting the comprehensiveness of our synthesis. To minimize time-lag bias and ensure inclusion of the most current evidence, we updated our search shortly before manuscript finalization on 22nd June 2025. One of the strengths of this umbrella review is the inclusion of grey literature, which helps to minimize the risk of publication bias. While many systematic reviews tend to focus solely on peer-reviewed sources, the incorporation of unpublished or non-indexed material increases the comprehensiveness and balance of our synthesis. A key strength of this review is the availability of a pre-defined protocol, which improves methodological transparency and minimizes the risk of selective outcome reporting. The use of a registered/published protocol also ensured that the conduct of the review remained consistent with its original objectives and methodology.

One limitation is that the conclusions are dependent on the quality and reporting standards of the included systematic reviews and meta-analyses, which were variable. This umbrella review relied on the data and interpretations provided by the included systematic reviews and meta-analyses, and therefore any inaccuracies or inconsistencies in those original reviews may have influenced the present findings [45, 46]. Furthermore, due to the nature of umbrella reviews, we were unable to assess or control for variations in primary study design, patient characteristics, or detailed intervention protocols beyond what was reported in the included reviews. For example, detailed patient characteristics such as history of periodontal surgery or specific lesion types, were not consistently reported across the included reviews. As such, this umbrella review could not analyse their impact on laser treatment outcomes.

Another key limitation of this umbrella review is that many of the included systematic reviews were rated as low quality. This undermines the overall certainty of the evidence and suggests that our conclusions, while supportive of laser therapy for DH, should be interpreted with caution. Until more high-quality, prospectively registered systematic reviews and meta-analyses are available, the strength of recommendations based on the current body of evidence remains limited.

Only systematic reviews published in English were included, as English is the only language spoken by multiple reviewers. Despite potential language bias, a decision was made at the start of the review that automatic translation tools would not be used for translation of potential review articles in languages other than English due to the possible inaccuracies in translation. Attempting to reduce the language bias, no language filter was applied in the search strategy.

### Directions for future research

Limited evidence exists for children and patients with MIH or post-bleaching sensitivity; (3) This review identifies several priority areas for future investigation: (1) **Standardization of protocols**: Uniform treatment parameters and validated outcome measures like VAS are needed to facilitate meaningful comparisons; (2) **Long-term efficacy**: Most studies focused on short-term outcomes; trials with follow-up beyond 12 months are essential to determine sustained benefit; (3) **Paediatric populations and specific conditions**: Limited evidence exists for children and patients with MIH or post-bleaching sensitivity; (3) **Head-to-head comparisons**: Direct comparisons between different laser types or laser versus established desensitizing agents will help refine clinical recommendations; (4) **Cost-effectiveness studies**: Health economic data are crucial to determine the viability of integrating laser therapy into standard care pathways.

While identifying the specific protocols associated with the best outcomes would be valuable, this level of detail requires trial-level analysis and is beyond the scope of this umbrella review. Future meta-analyses could address protocol-specific efficacy.

## Conclusions

This review consolidates evidence showing that laser therapy, either alone or in combination with desensitizing agents, may lead to a reduction in pain associated with DH. While short-term benefits appear more consistent, variability exists when lasers are compared with other desensitizing agents. However, the certainty of these conclusions is limited by methodological weaknesses and heterogeneity among existing systematic reviews. Thus, while laser therapy can be considered a potential non-invasive option, particularly when conventional treatments fail or are poorly tolerated, clinical judgment and shared decision-making remain essential when counselling patients.

The present findings highlight several key methodological gaps in the existing literature, such as inadequate protocol registration, poor reporting of bias assessment, and significant heterogeneity in outcome measures. From a policy perspective, this umbrella review underscores the need for standardized outcome measures and core outcome sets in future research to enable meaningful comparisons and improve evidence synthesis. The methodological limitations observed in this umbrella review should be factored into evidence grading and guideline formulation, especially when considering recommendations for the clinical use of laser therapy.

## Supplementary Information

Below is the link to the electronic supplementary material.

Supplementary Material 1

## Data Availability

No new primary data were generated or analyzed in this umbrella review. All data supporting the findings are derived from previously published systematic reviews, which are publicly available and cited throughout the manuscript. Summary data, including citation matrices, search strategies, and critical appraisal results, are provided in the appendices submitted with this article.
