# Supplementary material for: Effectiveness of lasers in managing dentine hypersensitivity: an umbrella review
Source: Lasers Med Sci. 2025 Oct 24;40(1):454. doi: 10.1007/s10103-025-04656-9 (PMC12552325; doi:10.1007/s10103-025-04656-9)
Supplement: Supplementary file 1 — Supplementary Material 1 [file 10103_2025_4656_MOESM1_ESM.docx]

Appendices

Appendix A

PRIOR Checklist

| **Section**  Topic | **#** | **Item** | **Location reported** |
| --- | --- | --- | --- |
| **TITLE** | | |  |
| Title | 1 | Identify the report as an overview of reviews. | 1 |
| **ABSTRACT** | | |  |
| Abstract | 2 | Provide a comprehensive and accurate summary of the purpose, methods, and results of the overview of reviews. | 1 |
| **INTRODUCTION** | | |  |
| Rationale | 3 | Describe the rationale for conducting the overview of reviews in the context of existing knowledge. | 2,3 |
| Objectives | 4 | Provide an explicit statement of the objective(s) or question(s) addressed by the overview of reviews. | 3 |
| **METHODS** | | |  |
| Eligibility criteria | 5a | Specify the inclusion and exclusion criteria for the overview of reviews. If supplemental primary studies were included, this should be stated, with a rationale. | 3,4 |
|  | 5b | Specify the definition of ‘systematic review’ as used in the inclusion criteria for the overview of reviews. | 3,4 |
| Information sources | 6 | Specify all databases, registers, websites, organizations, reference lists, and other sources searched or consulted to identify systematic reviews and supplemental primary studies (if included).  Specify the date when each source was last searched or consulted. | 3 |
| Search strategy | 7 | Present the full search strategies for all databases, registers and websites, such that they could be reproduced. Describe any search filters and limits applied. | A |
| Selection process | 8a | Describe the methods used to decide whether a systematic review or supplemental primary study (if included) met the inclusion criteria of the overview of reviews. | 4 |
|  | 8b | Describe how overlap in the populations, interventions, comparators, and/or outcomes of systematic reviews was identified and managed during study selection. | 4 |
| Data collection process | 9a | Describe the methods used to collect data from reports. | 4 |
|  | 9b | If applicable, describe the methods used to identify and manage primary study overlap at the level  of the comparison and outcome during data collection. For each outcome, specify the method used to illustrate and/or quantify the degree of primary study overlap across systematic reviews. | 4 |
|  | 9c | If applicable, specify the methods used to manage discrepant data across systematic reviews during data collection. | 4 |
| Data items | 10 | List and define all variables and outcomes for which data were sought. Describe any assumptions made and/or measures taken to identify and clarify missing or unclear information. | 4 |
| Risk of bias assessment | 11a | Describe the methods used to *assess* risk of bias or methodological quality of the included systematic reviews. | 5 |
|  | 11b | Describe the methods used to *collect* data on (from the systematic reviews) and/or *assess* the risk of bias of the primary studies included in the systematic reviews. Provide a justification for instances where flawed, incomplete, or missing assessments are identified but not re-assessed. | NA |
|  | 11c | Describe the methods used to *assess* the risk of bias of supplemental primary studies (if included). | NA |
| Synthesis methods | 12a | Describe the methods used to summarize or synthesize results and provide a rationale for the choice(s). | 6 |
|  | 12b | Describe any methods used to explore possible causes of heterogeneity among results. | NA |
|  | 12c | Describe any sensitivity analyses conducted to assess the robustness of the synthesized results. | NA |
| Reporting bias assessment | 13 | Describe the methods used to *collect* data on (from the systematic reviews) and/or *assess* the risk of bias due to missing results in a summary or synthesis (arising from reporting biases at the levels of the systematic reviews, primary studies, and supplemental primary studies, if included). | NA |
| Certainty assessment | 14 | Describe the methods used to *collect* data on (from the systematic reviews) and/or *assess* certainty (or confidence) in the body of evidence for an outcome. | NA |
| **RESULTS** | | |  |
| Systematic review and supplemental primary study selection | 15a | Describe the results of the search and selection process, including the number of records screened, assessed for eligibility, and included in the overview of reviews, ideally with a flow diagram. | 6,7 |
|  | 15b | Provide a list of studies that might appear to meet the inclusion criteria, but were excluded, with the main reason for exclusion. | 6 |

| **Section**  Topic | **#** | **Item** | **Location reported** |
| --- | --- | --- | --- |
| Characteristics of systematic reviews and supplemental primary studies | 16 | Cite each included systematic review and supplemental primary study (if included) and present its characteristics. | 8-28 |
| Primary study overlap | 17 | Describe the extent of primary study overlap across the included systematic reviews. | 29 |
| Risk of bias in systematic reviews, primary studies, and  supplemental primary studies | 18a | Present assessments of risk of bias or methodological quality for each included systematic review. | 29-31 |
|  | 18b | Present assessments (*collected* from systematic reviews or *assessed* anew) of the risk of bias of the primary studies included in the systematic reviews. | NA |
|  | 18c | Present assessments of the risk of bias of supplemental primary studies (if included). | NA |
| Summary or synthesis of results | 19a | For all outcomes, summarize the evidence from the systematic reviews and supplemental primary studies (if included). If meta-analyses were done, present for each the summary estimate and its precision and measures of statistical heterogeneity. If comparing groups, describe the direction of the effect. | 31,32 |
|  | 19b | If meta-analyses were done, present results of all investigations of possible causes of heterogeneity. | NA |
|  | 19c | If meta-analyses were done, present results of all sensitivity analyses conducted to assess the robustness of synthesized results. | NA |
| Reporting biases | 20 | Present assessments (*collected* from systematic reviews and/or *assessed* anew) of the risk of bias due to missing primary studies, analyses, or results in a summary or synthesis (arising from reporting biases at the levels of the systematic reviews, primary studies, and supplemental primary  studies, if included) for each summary or synthesis assessed. | NA |
| Certainty of evidence | 21 | Present assessments (*collected* or *assessed* anew) of certainty (or confidence) in the body of evidence for each outcome. | NA |
| **DISCUSSION** | | |  |
| Discussion | 22a | Summarize the main findings, including any discrepancies in findings across the included systematic reviews and supplemental primary studies (if included). | 32,33 |
|  | 22b | Provide a general interpretation of the results in the context of other evidence. | 32,33 |
|  | 22c | Discuss any limitations of the evidence from systematic reviews, their primary studies, and supplemental primary studies (if included) included in the overview of reviews. Discuss any limitations of the overview of reviews methods used. | 32,33,34 |
|  | 22d | Discuss implications for practice, policy, and future research (both systematic reviews and primary research). Consider the relevance of the findings to the end users of the overview of reviews, e.g., healthcare providers, policymakers, patients, among others. | 33,34 |
| **OTHER INFORMATION** | | |  |
| Registration and protocol | 23a | Provide registration information for the overview of reviews, including register name and registration number, or state that the overview of reviews was not registered. | 3 |
|  | 23b | Indicate where the overview of reviews protocol can be accessed, or state that a protocol was not prepared. | 3 |
|  | 23c | Describe and explain any amendments to information provided at registration or in the protocol. Indicate the stage of the overview of reviews at which amendments were made. | NA |
| Support | 24 | Describe sources of financial or non-financial support for the overview of reviews, and the role of the funders or sponsors in the overview of reviews. | 34 |
| Competing interests | 25 | Declare any competing interests of the overview of reviews' authors. | 34 |
| Author information | 26a | Provide contact information for the corresponding author. | TP |
|  | 26b | Describe the contributions of individual authors and identify the guarantor of the overview of reviews. | TP |
| Availability of data and other materials | 27 | Report which of the following are available, where they can be found, and under which conditions they may be accessed: template data collection forms; data collected from included systematic reviews and supplemental primary studies; analytic code; any other materials used in the overview of reviews. | A |

TP- Title Page, NA- Not Applicable, A-Appendices

**Appendix B**

**Descriptors used in the search query and search details for PubMed, Cochrane Library, Medline, Embase, Web of Science, Scopus, DARE, and PROSPERO and ProQuest (dissertations and theses) databases (inception to 3^rd^ February, 2025)**

| **Database** | **Search details** |
| --- | --- |
| **PubMed** | ("laser s"[All Fields] OR "lasers"[MeSH Terms] OR "lasers"[All Fields] OR "laser"[All Fields] OR "lasered"[All Fields] OR "lasering"[All Fields]) AND ("dentin"[MeSH Terms] OR "dentin"[All Fields] OR "dentine"[All Fields] OR "dentines"[All Fields] OR "dentins"[All Fields] OR "dentin s"[All Fields] OR "dentinal"[All Fields] OR "dentine s"[All Fields] OR ("dentin"[MeSH Terms] OR "dentin"[All Fields] OR "dentine"[All Fields] OR "dentines"[All Fields] OR "dentins"[All Fields] OR "dentin s"[All Fields] OR "dentinal"[All Fields] OR "dentine s"[All Fields]) OR ("dentin"[MeSH Terms] OR "dentin"[All Fields] OR "dentine"[All Fields] OR "dentines"[All Fields] OR "dentins"[All Fields] OR "dentin s"[All Fields] OR "dentinal"[All Fields] OR "dentine s"[All Fields]) OR ("dental health services"[MeSH Terms] OR ("dental"[All Fields] AND "health"[All Fields] AND "services"[All Fields]) OR "dental health services"[All Fields] OR "dental"[All Fields] OR "dentally"[All Fields] OR "dentals"[All Fields])) AND ("sensitive"[All Fields] OR "sensitively"[All Fields] OR "sensitives"[All Fields] OR "sensitivities"[All Fields] OR "sensitivity and specificity"[MeSH Terms] OR ("sensitivity"[All Fields] AND "specificity"[All Fields]) OR "sensitivity and specificity"[All Fields] OR "sensitivity"[All Fields] OR ("hypersensitiveness"[All Fields] OR "hypersensitivity"[MeSH Terms] OR "hypersensitivity"[All Fields] OR "hypersensitive"[All Fields] OR "hypersensitivities"[All Fields] OR "hypersensitization"[All Fields] OR "hypersensitize"[All Fields])) AND ("systematic review"[Publication Type] OR "systematic reviews as topic"[MeSH Terms] OR "systematic review"[All Fields] OR ("meta analysis"[Publication Type] OR "meta analysis as topic"[MeSH Terms] OR "meta analysis"[All Fields])) |
| **Cochrane Library** | #1 LASER  #2 DENTINE OR DENTIN OR DENTINAL OR DENTAL  #3 SENSITIVITY OR HYPERSENSITIVITY  #4 SYSTEMATIC REVIEW OR META ANALYSIS  #5 #1 AND #2 AND #3 AND #4 |
| **Medline** | 1. (Laser and Dental and sensitivity and review).mp. [mp=title, book title, abstract, original title, name of substance word, subject heading word, floating sub-heading word, keyword heading word, organism supplementary concept word, protocol supplementary concept word, rare disease supplementary concept word, unique identifier, synonyms, population supplementary concept word, anatomy supplementary concept word] 2. (Laser and Dental and sensitivity and metaanalysis).mp. [mp=title, book title, abstract, original title, name of substance word, subject heading word, floating sub-heading word, keyword heading word, organism supplementary concept word, protocol supplementary concept word, rare disease supplementary concept word, unique identifier, synonyms, population supplementary concept word, anatomy supplementary concept word] 3. (Laser and Dental and sensitivity and systematic review).mp. [mp=title, book title, abstract, original title, name of substance word, subject heading word, floating sub-heading word, keyword heading word, organism supplementary concept word, protocol supplementary concept word, rare disease supplementary concept word, unique identifier, synonyms, population supplementary concept word, anatomy supplementary concept word] 4. (Laser and Dentine and sensitivity and review).mp. [mp=title, book title, abstract, original title, name of substance word, subject heading word, floating sub-heading word, keyword heading word, organism supplementary concept word, protocol supplementary concept word, rare disease supplementary concept word, unique identifier, synonyms, population supplementary concept word, anatomy supplementary concept word] |
| **Scopus** | (TITLE-ABS-KEY("laser" OR "lasers" OR "lasered" OR "lasering")) AND (TITLE-ABS-KEY("dentin" OR "dentine" OR "dentinal" OR "dentines" OR "dentins" OR "dental"))  AND (TITLE-ABS-KEY("sensitive" OR "sensitivity" OR "hypersensitivity" OR "hypersensitiveness" OR "hypersensitive" OR "hypersensitization" OR "hypersensitize"))  AND (TITLE-ABS-KEY("systematic review" OR "meta analysis")) |
| **Embase** | #1 (laser* OR lasered OR lasering).mp.  #2 (dentin* OR dentine* OR dentinal OR dentins OR dentines).mp.  #3 (tooth* OR enamel* OR hard dental tissue* OR dental).mp.  #4 (sensitive OR sensitivity OR sensitivities OR hypersensitivity OR hypersensitiveness OR hypersensitive OR hypersensitization OR hypersensitize).mp.  #5 (systematic review OR meta analysis).mp.  #6 1 AND 2  #7 3 AND 6  #8 4 AND 7  #9 5 AND 8 |
| **Web of Science** | TS=("laser" OR "lasers" OR "lasered" OR "lasering") AND TS=("dentin" OR "dentine" OR "dentinal" OR "dentins" OR "dentines" OR "dental") AND TS=("sensitive" OR "sensitivity" OR "hypersensitive" OR "hypersensitivity" OR "hypersensitiveness" OR "hypersensitization" OR "hypersensitize") AND TS=("systematic review" OR "meta analysis") |
| **DARE** | ((((laser) AND ((((dentin) OR (dentine)) OR (dentinal)) OR (dental))) AND ((sensitivity) OR (hypersensitivity))) AND ((systematic review) OR (meta analysis))) IN DARE |
| **PROSPERO** | #1 LASER  #2 DENTIN OR DENTINE OR DENTINAL OR DENTAL  #3 SENSITIVITY OR HYPERSENSITIVITY  #4 SYSTEMATIC REVIEW OR META ANALYSIS  #5 #1 AND #2 AND #3 AND #4 |
| **ProQuest (Dissertations and Theses)** | (((laser) AND ((((dentin) OR (dentine)) OR (dentinal)) OR (dental))) AND ((sensitivity) OR (hypersensitivity))) AND ((systematic review) OR (meta analysis)) |

**Appendix C**

**JBI Data Extraction Tool for Systematic Reviews and Research Syntheses**


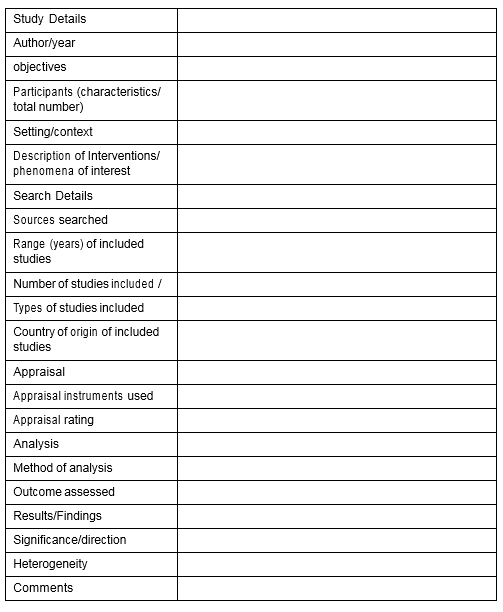


**Appendix D**

**JBI critical appraisal tool for Systematic Reviews and Research Syntheses**


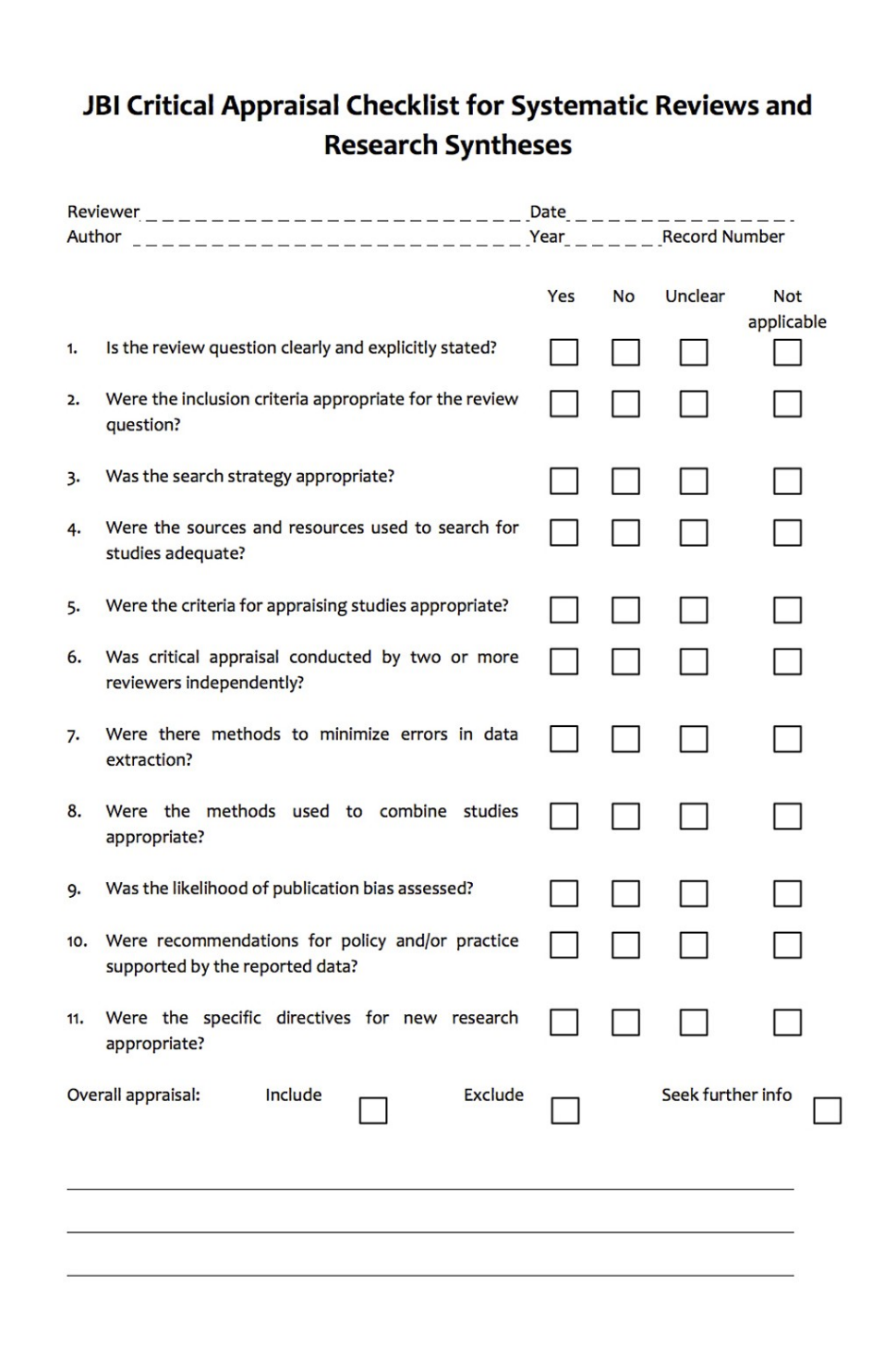


**Appendix E**

**Citation Matrix**

|  | **SYSTEMATIC REVIEWS** |  |  |  |  |  |  |  |  |  |  |  |  |  |  |  |  |  |  |  |  |  |  |  |  |  |
| --- | --- | --- | --- | --- | --- | --- | --- | --- | --- | --- | --- | --- | --- | --- | --- | --- | --- | --- | --- | --- | --- | --- | --- | --- | --- | --- |
| **PRIMARY STUDIES** | He et al/2011 | | Sgolastra et al/2011 | Sgolastra et al/2013 | Oliveira et al/2013 | Lin et al/2013 | West et al/2015 | Machado et al/2018 | Hu et al/2019 | Kong et al/2019 | Marto et al/2019 | Rezazadeh et al/2019 | Bellal et al/2021 | Mahdian et al/2021 | Zhou et al/2021 | Albar NH/2022 | AlHabdan et al/2022 | Baghani et al/2022 | Carneiro et al/2022 | Abdelkarim et al/2022 | Shakeel et al/2022 | Lestari et al/2023 | Pion et al/2023 | Cerqueira et al/2025 | Chen et al/2025 | Mohammadiann et al/2025 |
| Yilmaz et al/2011a (GaAIAs and Er,Cr:YSGG) |  |  | X | X | X |  |  | X | X | X | X | X | X |  |  | X |  |  | X | X |  | X |  |  |  | |
| Vieira et al/2009 | X | X | X | X | X |  | X | X | X |  |  |  | X | X |  |  |  |  |  |  |  | X |  |  |  | |
| Yilmaz et al/2011c (Diode laser vs NaF varnish) |  |  | X | X | X |  |  | X |  | X | X | X | X | X |  |  |  |  | X |  |  | X |  |  |  | |
| Yilmaz et al/2011b (Er,Cr:YSGG) |  |  | X | X | X |  |  | X | X | X | X |  | X |  |  | X |  |  |  |  |  | X |  |  |  | |
| Lier et al/2002 |  | X | X | X | X |  |  | X | X |  |  | X | X |  |  |  |  |  |  |  |  | X |  |  |  | |
| Birang et al/2007 |  | X | X | X |  |  |  | X | X |  | X | X |  |  |  |  |  |  |  |  |  | X |  |  |  | |
| Bal et al/2015 |  |  |  |  |  |  |  | X | X | X | X | X | X | X |  |  |  |  |  |  |  | X |  |  |  | |
| Orhan et al/2011 |  |  | X |  | X |  |  | X |  | X | X |  | X | X |  |  |  |  |  |  |  |  |  |  |  | |
| Schwarz et al/2002 | X |  | X |  | X | X |  | X |  |  |  |  |  |  |  |  |  |  |  |  |  | X |  |  |  | |
| Kara et al/2009 | X |  |  |  | X |  |  |  |  | X | X |  |  | X |  |  | X |  |  |  |  |  |  |  |  | |
| Sicilia et al/2009 | X |  | X |  | X |  |  | X |  |  | X |  |  |  |  |  |  |  | X |  |  |  |  |  |  | |
| Aranha and Eduardo/2012 |  |  | X |  | X |  |  |  | X | X | X |  |  |  |  | X |  |  |  |  |  |  |  |  |  | |
| Gerschman et al/1994 |  |  | X |  | X | X |  | X |  |  |  |  | X |  |  |  |  |  |  |  |  |  |  |  |  | |
| Gentile and Greghi/2004 |  |  | X |  | X |  |  | X |  |  |  |  | X |  |  |  |  |  |  | X |  |  |  |  |  | |
| Ipci et al/2009 | X |  |  | X | X |  |  |  |  |  | X |  |  |  |  |  |  |  |  |  |  | X |  |  |  | |
| Dilsiz et al/2010a |  |  |  |  | X |  |  | X | X | X | X |  |  |  |  |  |  |  |  |  |  |  |  |  |  | |
| Ehlers et al/2012 |  |  |  |  |  | X |  |  |  | X | X |  |  |  | X |  |  |  |  |  |  | X |  |  |  | |
| Femiano et al/2013 |  |  |  |  |  | X |  |  |  | X | X |  |  |  | X |  |  |  |  |  |  | X |  |  |  | |
| Lopes et al/2013 |  |  |  |  |  |  |  |  |  | X | X |  |  |  | X |  |  |  |  |  | X | X |  |  |  | |
| Yilmaz et al/2014 |  |  |  |  |  |  |  | X | X |  | X |  | X |  |  | X |  |  |  |  |  |  |  |  |  | |
| Lopes et al/2015 |  |  |  |  |  |  | X |  |  | X | X |  |  |  | X |  |  |  |  |  |  | X |  |  |  | |
| Soares et al/2016 |  |  |  |  |  |  |  |  | X | X | X |  |  |  |  |  | X |  |  | X |  |  |  |  |  | |
| Lopes et al/2017 |  |  |  |  |  |  |  |  |  | X |  |  |  |  | X |  | X |  | X |  |  | X |  |  |  | |
| Maximiano et al/2019 |  |  |  |  |  |  |  |  |  |  |  | X | X |  |  |  | X |  |  |  | X |  |  | X |  | |
| Tengrungsun & Sangkla/2008 | X |  |  |  | X | X |  |  |  |  | X |  |  |  |  |  |  |  |  |  |  |  |  |  |  | |
| Aranha et al/2009 |  |  |  | X |  |  |  |  |  |  | X |  |  |  | X |  |  |  |  |  |  | X |  |  |  | |
| Flecha et al/2013 |  |  |  |  |  | X |  |  |  |  | X |  | X |  |  |  |  |  |  |  |  | X |  |  |  | |
| Talesara et al/2014 |  |  |  |  |  | X |  |  |  | X |  |  |  |  |  |  | X |  |  |  |  | X |  |  |  | |
| Ozlem et al/2018 |  |  |  |  |  |  |  |  |  |  |  |  |  |  | X | X |  |  |  |  | X | X |  |  |  | |
| Suri et al/2016 |  |  |  |  |  |  |  |  |  |  | X |  | X | X |  |  |  |  |  |  |  |  |  |  | X | |
| Chebel et al/2018 |  |  |  |  |  |  |  |  |  |  |  |  |  | X |  |  | X |  |  |  |  | X |  | X |  | |
| Guo et al/2019 |  |  |  |  |  |  |  |  |  |  |  |  |  | X |  |  | X |  |  |  | X |  |  | X |  | |
| Kumar et al/ 2005 | X |  |  |  |  |  |  |  |  |  |  |  |  | X |  |  | X |  |  |  |  |  |  |  |  | |
| Dilsiz et al/2010b |  |  |  |  | X |  |  |  |  | X | X |  |  |  |  |  |  |  |  |  |  |  |  |  |  | |
| Lund et al/2013 |  |  |  |  |  |  |  |  |  |  | X |  | X |  |  |  |  |  |  |  |  | X |  |  |  | |
| Ko et al/2014 |  |  |  |  |  | X |  |  |  | X | X |  |  |  |  |  |  |  |  |  |  |  |  |  |  | |
| Moosavi et al/2016 |  |  |  |  |  |  |  | X |  |  | X |  |  |  |  |  |  | X |  |  |  |  |  |  |  | |
| Corona et al/2003 | X |  |  |  | X |  |  |  |  |  |  |  |  |  |  |  |  |  |  |  |  |  |  | X |  | |
| Narayanan et al/2019 |  |  |  |  |  |  |  |  |  |  |  |  |  |  |  |  |  |  | X |  |  | X |  | X |  | |
| Ciaramicoli et al/2003 |  |  |  | X |  |  |  |  |  |  |  |  |  |  |  |  |  |  |  |  |  | X |  |  |  | |
| Duran and Sengun/2004 |  |  |  | X | X |  |  |  |  |  |  |  |  |  |  |  |  |  |  |  |  |  |  |  |  | |
| Polderman and Frencken/2007 |  |  |  | X | X |  |  |  |  |  |  |  |  |  |  |  |  |  |  |  |  |  |  |  |  | |
| Ozen et al/2009 |  |  |  |  | X |  |  |  |  | X |  |  |  |  |  |  |  |  |  |  |  |  |  |  |  | |
| Erdemir et al/2010 |  |  |  |  | X |  |  |  |  | X |  |  |  |  |  |  |  |  |  |  |  |  |  |  |  | |
| Sethna et al/2011 |  |  |  | X |  |  |  |  |  | X |  |  |  |  |  |  |  |  |  |  |  |  |  |  |  | |
| Abed et al/2012 |  |  |  |  |  |  |  |  |  |  |  |  |  | X |  |  |  |  |  |  | X |  |  |  |  | |
| Umberto et al/2012 |  |  |  |  |  |  |  |  |  |  |  |  |  |  |  |  |  |  | X | X |  |  |  |  |  | |
| Lee et al/2015 |  |  |  |  |  |  |  |  |  |  | X |  | X |  |  |  |  |  |  |  |  |  |  |  |  | |
| Dantas et al/2016 |  |  |  |  |  |  |  |  |  | X | X |  |  |  |  |  |  |  |  |  |  |  |  |  |  | |
| Garcia et al/2017 |  |  |  |  |  |  |  | X |  |  |  |  | X |  |  |  |  |  |  |  |  |  |  |  |  | |
| Maximiano et al/2018 |  |  |  |  |  |  |  |  | X |  |  |  |  | X |  |  |  |  |  |  |  |  |  |  |  | |
| Osmari et al/2018 |  |  |  |  |  |  |  |  |  | X |  |  |  | X |  |  |  |  |  |  |  |  |  |  |  | |
| Raut et al/2018 |  |  |  |  |  |  |  | X |  |  |  |  |  |  |  |  |  |  | X |  |  |  |  |  |  | |
| Moura et al/2019 |  |  |  |  |  |  |  |  |  |  |  |  |  |  |  |  |  |  | X |  |  | X |  |  |  | |
| Pourshahidi et al/2019 |  |  |  |  |  |  |  |  |  |  |  |  |  |  |  | X |  |  | X |  |  |  |  |  |  | |
| Naghsh et al/2020 |  |  |  |  |  |  |  |  |  |  |  |  | X |  |  |  |  |  | X |  |  |  |  |  |  | |
| Oliveira et al/2018 |  |  |  |  |  |  |  |  |  |  |  |  |  |  |  |  |  |  |  |  |  | X |  | X |  | |
| Ortiz et al/2019 |  |  |  |  |  |  |  |  |  |  |  |  | X |  |  |  |  |  |  |  |  |  |  | X |  | |
| Moeintaghavi et al/2021 |  |  |  |  |  |  |  |  |  |  |  |  |  |  |  | X |  |  |  |  |  |  |  | X |  | |
| Zhao et al/2023 |  |  |  |  |  |  |  |  |  |  |  |  |  |  |  |  |  |  |  |  |  |  | X | X |  | |
| Tarbet et al/1979 |  |  |  |  | X |  |  |  |  |  |  |  |  |  |  |  |  |  |  |  |  |  |  |  |  | |
| McBride et al/1991 |  |  |  |  | X |  |  |  |  |  |  |  |  |  |  |  |  |  |  |  |  |  |  |  |  | |
| Dondi dall’Orologio & Malferrari/1993 |  |  |  |  | X |  |  |  |  |  |  |  |  |  |  |  |  |  |  |  |  |  |  |  |  | |
| Dunne & Hannington-Kiff/1993 |  |  |  |  | X |  |  |  |  |  |  |  |  |  |  |  |  |  |  |  |  |  |  |  |  | |
| Gelskey et al/1993 |  |  |  |  |  |  |  |  |  |  |  |  |  |  |  |  |  |  |  |  |  | X |  |  |  | |
| Lan et al/1996 |  |  |  |  |  |  |  |  |  |  |  |  |  |  |  |  |  |  |  |  |  | X |  |  |  | |
| Gutknecht et al/1997 |  |  |  |  |  |  |  |  |  |  |  |  |  |  |  |  |  |  |  |  |  | X |  |  |  | |
| Ide et al/1998 |  |  |  |  | X |  |  |  |  |  |  |  |  |  |  |  |  |  |  |  |  |  |  |  |  | |
| Moritz et al/1998 |  |  |  |  |  |  |  |  |  |  |  |  |  |  |  |  |  |  |  |  |  | X |  |  |  | |
| Yates et al/1998 |  |  |  |  | X |  |  |  |  |  |  |  |  |  |  |  |  |  |  |  |  |  |  |  |  | |
| Zhang et al/1998 |  |  |  |  |  |  |  |  |  |  |  |  |  |  |  |  |  |  |  |  |  | X |  |  |  | |
| Morris et al/1999 |  |  |  |  | X |  |  |  |  |  |  |  |  |  |  |  |  |  |  |  |  |  |  |  |  | |
| Marsilio et al/2003 |  |  |  |  |  |  |  |  |  |  |  |  |  |  |  |  |  |  |  | X |  |  |  |  |  | |
| Frechoso et al/2003 |  |  |  |  | X |  |  |  |  |  |  |  |  |  |  |  |  |  |  |  |  |  |  |  |  | |
| Zhang et al/2003 |  |  |  |  | X |  |  |  |  |  |  |  |  |  |  |  |  |  |  |  |  |  |  |  |  | |
| Gillam et al/2004 |  |  |  |  | X |  |  |  |  |  |  |  |  |  |  |  |  |  |  |  |  |  |  |  |  | |
| Ladalardo et al/2004 |  |  |  |  |  |  | X |  |  |  |  |  |  |  |  |  |  |  |  |  |  |  |  |  |  | |
| Kakaboura et al/2005 |  |  |  |  | X |  |  |  |  |  |  |  |  |  |  |  |  |  |  |  |  |  |  |  |  | |
| Pamir et al/2005 |  |  |  |  | X |  |  |  |  |  |  |  |  |  |  |  |  |  |  |  |  |  |  |  |  | |
| Singal et al/2005 |  |  |  | X |  |  |  |  |  |  |  |  |  |  |  |  |  |  |  |  |  |  |  |  |  | |
| Zantner et al/2006 |  |  |  |  | X |  |  |  |  |  |  |  |  |  |  |  |  |  |  |  |  |  |  |  |  | |
| Lizarelli et al/2007 |  |  |  |  |  |  |  |  |  |  |  |  | X |  |  |  |  |  |  |  |  |  |  |  |  | |
| Du Min et al/2008 |  |  |  |  |  |  |  |  |  | X |  |  |  |  |  |  |  |  |  |  |  |  |  |  |  | |
| Kobler et al/2008 |  |  |  | X |  |  |  |  |  |  |  |  |  |  |  |  |  |  |  |  |  |  |  |  |  | |
| Marson et al/2008 |  |  |  |  |  |  |  |  |  |  | X |  |  |  |  |  |  |  |  |  |  |  |  |  |  | |
| Azarpazhooh et al/2009 |  |  |  |  |  |  |  |  |  | X |  |  |  |  |  |  |  |  |  |  |  |  |  |  |  | |
| Clavijo et al/2009 |  |  |  | X |  |  |  |  |  |  |  |  |  |  |  |  |  |  |  |  |  |  |  |  |  | |
| Dilsiz et al/2009 |  |  | X |  |  |  |  |  |  |  |  |  |  |  |  |  |  |  |  |  |  |  |  |  |  | |
| Hamlin et al/2009 |  |  |  |  | X |  |  |  |  |  |  |  |  |  |  |  |  |  |  |  |  |  |  |  |  | |
| Hoang-Dao et al/2009 |  |  |  |  |  |  |  |  |  | X |  |  |  |  |  |  |  |  |  |  |  |  |  |  |  | |
| Schiff et al/2009 |  |  |  |  | X |  |  |  |  |  |  |  |  |  |  |  |  |  |  |  |  |  |  |  |  | |
| Eitner et al/2010 |  |  |  |  | X |  |  |  |  |  |  |  |  |  |  |  |  |  |  |  |  |  |  |  |  | |
| Genovesi et al/2010 |  |  |  |  |  |  |  |  |  |  | X |  |  |  |  |  |  |  |  |  |  |  |  |  |  | |
| Gurgan et al/2010 |  |  |  |  |  |  |  |  |  |  | X |  |  |  |  |  |  |  |  |  |  |  |  |  |  | |
| Hughes et al/2010 |  |  |  |  |  |  |  |  |  | X |  |  |  |  |  |  |  |  |  |  |  |  |  |  |  | |
| Jalali et al/2010 |  |  |  |  |  |  |  |  |  | X |  |  |  |  |  |  |  |  |  |  |  |  |  |  |  | |
| Litkowski et al/2010 |  |  |  |  |  |  |  |  |  | X |  |  |  |  |  |  |  |  |  |  |  |  |  |  |  | |
| Mason et al/2010 |  |  |  |  |  |  |  |  |  | X |  |  |  |  |  |  |  |  |  |  |  |  |  |  |  | |
| Pesevska et al/2010 |  |  |  |  |  |  |  |  |  |  | X |  |  |  |  |  |  |  |  |  |  |  |  |  |  | |
| Pradeep et al/2010 |  |  |  |  |  |  |  |  |  | X |  |  |  |  |  |  |  |  |  |  |  |  |  |  |  | |
| Salian et al/2010 |  |  |  |  |  |  |  |  |  | X |  |  |  |  |  |  |  |  |  |  |  |  |  |  |  | |
| Sharma et al/2010 |  |  |  |  |  |  |  |  |  | X |  |  |  |  |  |  |  |  |  |  |  |  |  |  |  | |
| Shetty et al/2010 |  |  |  |  | X |  |  |  |  |  |  |  |  |  |  |  |  |  |  |  |  |  |  |  |  | |
| Abed et al/2011 |  |  |  |  |  |  |  |  |  |  |  |  |  |  |  |  | X |  |  |  |  |  |  |  |  | |
| Assis et al/2011 |  |  |  |  | X |  |  |  |  |  |  |  |  |  |  |  |  |  |  |  |  |  |  |  |  | |
| Brahmbhatt et al/2011 |  |  |  | X |  |  |  |  |  |  |  |  |  |  |  |  |  |  |  |  |  |  |  |  |  | |
| Castillo et al/2011 (Lima, Peru) |  |  |  |  | X |  |  |  |  |  |  |  |  |  |  |  |  |  |  |  |  |  |  |  |  | |
| Castillo et al/2011 (Cusco, Peru) |  |  |  |  | X |  |  |  |  |  |  |  |  |  |  |  |  |  |  |  |  |  |  |  |  | |
| He et al/2011 (Na monofluorophosphate vs SnF) |  |  |  |  |  |  |  |  |  | X |  |  |  |  |  |  |  |  |  |  |  |  |  |  |  | |
| He et al/2011 (Stannous containing NaF vs Ar, CaCO3,F) |  |  |  |  |  |  |  |  |  | X |  |  |  |  |  |  |  |  |  |  |  |  |  |  |  | |
| Kossatz et al/2011 |  |  |  |  |  |  |  |  |  |  | X |  |  |  |  |  |  |  |  |  |  |  |  |  |  | |
| Tirapelli et al/2011 |  |  |  |  |  |  |  |  |  | X |  |  |  |  |  |  |  |  |  |  |  |  |  |  |  | |
| Won et al/2011 |  |  |  |  |  |  |  | X |  |  |  |  |  |  |  |  |  |  |  |  |  |  |  |  |  | |
| Brahmbhatt et al/2012 |  |  |  |  |  |  |  |  |  | X |  |  |  |  |  |  |  |  |  |  |  |  |  |  |  | |
| Correa et al/2012 |  |  |  |  |  |  |  | X |  |  |  |  |  |  |  |  |  |  |  |  |  |  |  |  |  | |
| De Almeida et al/2012 |  |  |  |  |  |  |  |  |  |  | X |  |  |  |  |  |  |  |  |  |  |  |  |  |  | |
| Drebenstedt et al/2012 |  |  |  | X |  |  |  |  |  |  |  |  |  |  |  |  |  |  |  |  |  |  |  |  |  | |
| Guentsch et al/2012 |  |  |  |  |  |  |  |  |  | X |  |  |  |  |  |  |  |  |  |  |  |  |  |  |  | |
| Kashyap et al/2012 |  |  |  |  |  |  |  |  |  | X |  |  |  |  |  |  |  |  |  |  |  |  |  |  |  | |
| Mogharehabed et al/2012 |  |  |  |  |  |  |  |  |  |  | X |  |  |  |  |  |  |  |  |  |  |  |  |  |  | |
| Pradeep et al/2012 |  |  |  |  |  |  |  |  |  | X |  |  |  |  |  |  |  |  |  |  |  |  |  |  |  | |
| Raichur et al/2012 |  |  |  |  |  |  |  |  |  | X |  |  |  |  |  |  |  |  |  |  |  |  |  |  |  | |
| Talesara et al/2012 |  |  |  |  |  |  |  |  |  |  |  |  |  | X |  |  |  |  |  |  |  |  |  |  |  | |
| Vora et al/2012 |  |  |  |  |  |  |  |  |  | X |  |  |  |  |  |  |  |  |  |  |  |  |  |  |  | |
| Bao et al/2013 |  |  |  |  |  |  |  | X |  |  |  |  |  |  |  |  |  |  |  |  |  |  |  |  |  | |
| Gibson et al/2013 |  |  |  |  |  |  |  |  |  | X |  |  |  |  |  |  |  |  |  |  |  |  |  |  |  | |
| Kumari et al/2013 |  |  |  |  |  |  |  |  |  | X |  |  |  |  |  |  |  |  |  |  |  |  |  |  |  | |
| Raichur et al/2013 |  |  |  |  |  |  |  |  |  |  | X |  |  |  |  |  |  |  |  |  |  |  |  |  |  | |
| Sharma et al/2013 |  |  |  |  |  |  |  |  |  | X |  |  |  |  |  |  |  |  |  |  |  |  |  |  |  | |
| West et al/2013 |  |  |  |  |  |  |  |  |  | X |  |  |  |  |  |  |  |  |  |  |  |  |  |  |  | |
| Ye et al/2013 |  |  |  |  |  |  |  | X |  |  |  |  |  |  |  |  |  |  |  |  |  |  |  |  |  | |
| Antoniazzi et al/2014 |  |  |  |  |  |  |  |  |  | X |  |  |  |  |  |  |  |  |  |  |  |  |  |  |  | |
| De Almeida Farhat et al/2014 |  |  |  |  |  |  |  |  |  |  | X |  |  |  |  |  |  |  |  |  |  |  |  |  |  | |
| Doshi et al/2014 |  |  |  |  |  |  |  |  |  |  |  |  | X |  |  |  |  |  |  |  |  |  |  |  |  | |
| Ding et al/2014 |  |  |  |  |  |  |  |  |  | X |  |  |  |  |  |  |  |  |  |  |  |  |  |  |  | |
| Hashim et al/2014 |  |  |  |  |  |  |  |  |  |  | X |  |  |  |  |  |  |  |  |  |  |  |  |  |  | |
| Mehta et al/2014 |  |  |  |  |  |  |  |  |  | X |  |  |  |  |  |  |  |  |  |  |  |  |  |  |  | |
| Torres et al/2014 |  |  |  |  |  |  |  |  |  | X |  |  |  |  |  |  |  |  |  |  |  |  |  |  |  | |
| Vano et al/2014 |  |  |  |  |  |  |  |  |  | X |  |  |  |  |  |  |  |  |  |  |  |  |  |  |  | |
| Franca et al/2015 |  |  |  |  |  |  |  |  |  | X |  |  |  |  |  |  |  |  |  |  |  |  |  |  |  | |
| Konekeri et al/2015 |  |  |  |  |  |  |  |  |  | X |  |  |  |  |  |  |  |  |  |  |  |  |  |  |  | |
| Mehta et al/2015 |  |  |  |  |  |  |  |  |  | X |  |  |  |  |  |  |  |  |  |  |  |  |  |  |  | |
| Moosavi et al/2015 |  |  |  |  |  |  |  |  |  |  | X |  |  |  |  |  |  |  |  |  |  |  |  |  |  | |
| Patil et al/2015 |  |  |  |  |  |  |  |  |  | X |  |  |  |  |  |  |  |  |  |  |  |  |  |  |  | |
| Parkinson et al/2015 |  |  |  |  |  |  |  |  |  | X |  |  |  |  |  |  |  |  |  |  |  |  |  |  |  | |
| Pinna et al/2015 |  |  |  |  |  |  |  |  |  | X |  |  |  |  |  |  |  |  |  |  |  |  |  |  |  | |
| Yaghini et al/2015 |  |  |  |  |  |  |  |  |  |  | X |  |  |  |  |  |  |  |  |  |  |  |  |  |  | |
| Dias et al/2016 |  |  |  |  |  |  |  |  |  |  |  |  |  |  |  |  |  | X |  |  |  |  |  |  |  | |
| Haluk et al/2016 |  |  |  |  |  |  |  |  |  |  |  |  |  |  | X |  |  |  |  |  |  |  |  |  |  | |
| Katanec et al/2016 |  |  |  |  |  |  |  |  |  | X |  |  |  |  |  |  |  |  |  |  |  |  |  |  |  | |
| Kumari et al/2016 |  |  |  |  |  |  |  |  |  | X |  |  |  |  |  |  |  |  |  |  |  |  |  |  |  | |
| Majji et al/2016 |  |  |  |  |  |  |  |  |  | X |  |  |  |  |  |  |  |  |  |  |  |  |  |  |  | |
| Parkinson et al/2016 |  |  |  |  |  |  |  |  |  | X |  |  |  |  |  |  |  |  |  |  |  |  |  |  |  | |
| Wang et al/2016 |  |  |  |  |  |  |  |  |  | X |  |  |  |  |  |  |  |  |  |  |  |  |  |  |  | |
| Young et al/2016 |  |  |  |  |  |  |  |  |  | X |  |  |  |  |  |  |  |  |  |  |  |  |  |  |  | |
| Zang et al/2016 |  |  |  |  |  |  |  |  |  | X |  |  |  |  |  |  |  |  |  |  |  |  |  |  |  | |
| Agrawal et al/2017 |  |  |  |  |  |  |  |  |  |  |  |  |  |  |  |  |  |  |  | X |  |  |  |  |  | |
| Calheiros et al/2017 |  |  |  |  |  |  |  |  |  |  |  |  |  |  |  |  |  | X |  |  |  |  |  |  |  | |
| Canali et al/2017 |  |  |  |  |  |  |  |  |  | X |  |  |  |  |  |  |  |  |  |  |  |  |  |  |  | |
| Hall et al/2017 |  |  |  |  |  |  |  |  |  | X |  |  |  |  |  |  |  |  |  |  |  |  |  |  |  | |
| Han et al/2017 |  |  |  |  |  |  |  |  |  | X |  |  |  |  |  |  |  |  |  |  |  |  |  |  |  | |
| Idon et al/2017 |  |  |  |  |  |  |  |  |  | X |  |  |  |  |  |  |  |  |  |  |  |  |  |  |  | |
| Lima et al/2017 |  |  |  |  |  |  |  |  |  |  |  |  |  |  |  |  |  |  |  |  |  | X |  |  |  | |
| Madruga et al/2017 |  |  |  |  |  |  |  |  |  | X |  |  |  |  |  |  |  |  |  |  |  |  |  |  |  | |
| Tevatia et al/2017 |  |  |  |  |  |  |  |  |  |  |  |  | X |  |  |  |  |  |  |  |  |  |  |  |  | |
| Zheng et al/2017 |  |  |  |  |  |  |  | X |  |  |  |  |  |  |  |  |  |  |  |  |  |  |  |  |  | |
| Alencar et al/2018 |  |  |  |  |  |  |  |  |  |  |  |  |  |  |  |  |  | X |  |  |  |  |  |  |  | |
| Amaechi et al/2018 |  |  |  |  |  |  |  |  |  | X |  |  |  |  |  |  |  |  |  |  |  |  |  |  |  | |
| Kumar et al/2018 |  |  |  |  |  |  |  |  |  | X |  |  |  |  |  |  |  |  |  |  |  |  |  |  |  | |
| Praveen et al/2018 |  |  |  |  |  |  |  |  |  |  |  |  |  |  |  |  |  |  |  |  |  | X |  |  |  | |
| Ravishankar et al/2018 |  |  |  |  |  |  |  |  |  | X |  |  |  |  |  |  |  |  |  |  |  |  |  |  |  | |
| Seong et al/2018 |  |  |  |  |  |  |  |  |  | X |  |  |  |  |  |  |  |  |  |  |  |  |  |  |  | |
| Tabatabaei et al/2018 |  |  |  |  |  |  |  |  |  |  |  |  |  |  |  |  |  |  |  |  |  | X |  |  |  | |
| Tabibzadeh et al/2018 |  |  |  |  |  |  |  |  |  |  |  |  |  |  |  |  |  |  | X |  |  |  |  |  |  | |
| Vano et al/2018 |  |  |  |  |  |  |  |  |  | X |  |  |  |  |  |  |  |  |  |  |  |  |  |  |  | |
| El Mobadder et al/2019 |  |  |  |  |  |  |  |  |  |  |  |  |  |  |  |  |  |  |  |  |  | X |  |  |  | |
| De Paula et al/2019 |  |  |  |  |  |  |  |  |  |  |  |  |  |  |  |  |  | X |  |  |  |  |  |  |  | |
| Vieira et al/2019 |  |  |  |  |  |  |  |  |  |  |  |  |  |  |  |  |  |  |  |  |  |  | X |  |  | |
| Yadav et al/2019 |  |  |  |  |  |  |  |  |  |  |  |  |  |  |  |  |  |  |  |  |  | X |  |  |  | |
| Alencar et al/2020 |  |  |  |  |  |  |  |  |  |  |  |  | X |  |  |  |  |  |  |  |  |  |  |  |  | |
| De Silva et al/2020 |  |  |  |  |  |  |  |  |  |  |  |  |  |  |  |  |  | X |  |  |  |  |  |  |  | |
| Miron et al/2020 |  |  |  |  |  |  |  |  |  |  |  |  |  |  |  |  |  |  |  |  | X |  |  |  |  | |
| Muniz et al/2020 |  |  |  |  |  |  |  |  |  |  |  |  |  |  |  |  |  |  |  |  |  |  | X |  |  | |
| Pantuzzo et al/2020 |  |  |  |  |  |  |  |  |  |  |  |  | X |  |  |  |  |  |  |  |  |  |  |  |  | |
| Sobral et al/2021 |  |  |  |  |  |  |  |  |  |  |  |  |  |  |  |  |  |  |  |  |  |  | X |  |  | |
| Pion et al/2022 |  |  |  |  |  |  |  |  |  |  |  |  |  |  |  |  |  |  |  |  |  |  | X |  |  | |
| Fosatti et al/2023 |  |  |  |  |  |  |  |  |  |  |  |  |  |  |  |  |  |  |  |  |  |  | X |  |  | |
| Mandetta et al/2023 |  |  |  |  |  |  |  |  |  |  |  |  |  |  |  |  |  |  |  |  |  |  | X |  |  | |
| Tasanee et al/2008 |  |  |  |  |  |  |  |  |  |  |  |  |  |  |  |  |  |  |  |  |  |  |  | X |  | |
| Alessandra et al/2009 |  |  |  |  |  |  |  |  |  |  |  |  |  |  |  |  |  |  |  |  |  |  |  | X |  | |
| Cankat et al/2009 |  |  |  |  |  |  |  |  |  |  |  |  |  |  |  |  |  |  |  |  |  |  |  | X |  | |
| Sebnwm et al/2009 |  |  |  |  |  |  |  |  |  |  |  |  |  |  |  |  |  |  |  |  |  |  |  | X |  | |
| Hasan et al/2011 |  |  |  |  |  |  |  |  |  |  |  |  |  |  |  |  |  |  |  |  |  |  |  | X |  | |
| Ahmad et al/2012 |  |  |  |  |  |  |  |  |  |  |  |  |  |  |  |  |  |  |  |  |  |  |  | X |  | |
| Muniz et al/2019 |  |  |  |  |  |  |  |  |  |  |  |  |  |  |  |  |  |  |  |  |  |  |  | X |  | |
| Bhavsar BA et al/2020 |  |  |  |  |  |  |  |  |  |  |  |  |  |  |  |  |  |  |  |  |  |  |  | X |  | |
| Jain et al/2020 |  |  |  |  |  |  |  |  |  |  |  |  |  |  |  |  |  |  |  |  |  |  |  |  | X | |
| Sgreccia P et al/2020 |  |  |  |  |  |  |  |  |  |  |  |  |  |  |  |  |  |  |  |  |  |  |  | X |  | |
| Sgreccia PC et al/2020 |  |  |  |  |  |  |  |  |  |  |  |  |  |  |  |  |  |  |  |  |  |  |  | X |  | |
| Hu et al/2021 |  |  |  |  |  |  |  |  |  |  |  |  |  |  |  |  |  |  |  |  |  |  |  | X |  | |
| Zhao et al/2021 |  |  |  |  |  |  |  |  |  |  |  |  |  |  |  |  |  |  |  |  |  |  |  | X |  | |
| Forouzande et al/2022 |  |  |  |  |  |  |  |  |  |  |  |  |  |  |  |  |  |  |  |  |  |  |  | X |  | |
| Tolentino et al/2022 |  |  |  |  |  |  |  |  |  |  |  |  |  |  |  |  |  |  |  |  |  |  |  | X |  | |
| Yahya et al/2022 |  |  |  |  |  |  |  |  |  |  |  |  |  |  |  |  |  |  |  |  |  |  |  |  | X | |
| Narges et al/2024 |  |  |  |  |  |  |  |  |  |  |  |  |  |  |  |  |  |  |  |  |  |  |  | X |  | |
| Sajith et al/2024 |  |  |  |  |  |  |  |  |  |  |  |  |  |  |  |  |  |  |  |  |  |  |  | X |  | |

**Appendix F**

**ROBIS Tool with Comments**

|  | **PHASE 2** | | | | **PHASE 3** |  | |
| --- | --- | --- | --- | --- | --- | --- | --- |
| **Systematic review** | **1. Study Eligibility Criteria** | **2. Identification and Selection of Studies** | **3.Data Collection and Study Appraisal** | **4. Synthesis and Findings** | **Risk of Bias in the Review** | **Comments** |  |
| Chen et al/2025 | Y | Y | Y | Y |  |  |  |
| Mahdian et al/ 2021 | Y | Y | Y | Y |  |  |  |
| Hu et al/ 2019 | N | PY | Y | Y |  | 1.1-Protocol Comparator: "Placebo or NaF" Published Review Comparator: "Placebo or no treatment" NaF studies were excluded, and "no treatment" was added without clear justification. |  |
| Kong et al/ 2019 | N | Y | Y | N |  | 1.1- Protocol - Comparator, intervention and outcome assessed differ from the published review.  4.4- Heterogeneity mentioned in Methodology but not reported in Results. |  |
| Bellal et al/ 2021 | N | Y | PN | Y |  | 1.1-Protocol Comparator: "lasers vs NaF" and "lasers vs Placebo or no treatment" Published Review Comparator: "lasers vs Placebo or no treatment" NaF studies were excluded without clear justification. 1.3- The eligibility states “lasers vs placebo,” but the objective and included studies focus only on near-infrared lasers. (lasers is a broad term, should be more specific if including only one type) 3.5- No. of reviewers for RoB unclear/not explicitly stated. |  |
| Albar NH/ 2022 | PY | Y | Y | N |  | 1.3- In Protocol- mentioned using VAS under outcome assessed, but in Final Review VAS was not mentioned in methods section under eligibility criteria. (was mentioned in Results section) 4.4- Heterogeneity was not assessed 4.5- No funnel plot or sensitivity analysis performed (although they stated that publication bias was "not serious" in Table 3) |  |
| Baghani and Karrabi/ 2022 | N | N | Y | Y |  | 1.1- No Protocol  2.2- No additional methods used (manual search/ref. search/contacting authors/ searching institutional repositories) 2.3- Full search strategy not given |  |
| He et al/ 2011 | N | N | Y | N |  | 1.1- No Protocol  2.3- Full search strategy not given 4.5- No funnel plot or sensitivity analysis performed |  |
| Sgolastra et al/  2013 | N | N | Y | N |  | 1.1- No Protocol  1.2- inclusion criteria addressed effectiveness but did not explicitly include studies assessing safety, despite it being a stated secondary aim. 2.3- Full search strategy not given 4.5- No funnel plot or sensitivity analysis performed |  |
| Machado et al/ 2018 | N | N | N | N |  | 1.1- No Protocol  1.2/1.3- under objective and title PBM is used but in search terms low level laser is used not PBM. 2.1- only one database searched  2.3--lacks explicit MeSH terms, field tags, and reproducibility details, Full search strategy not given 3.1- Only one author performed data collection 3.4-RoB not reported in results/discussion (only in methods) 4.2- Cost effectivity analysis not performed (stated under secondary objective), no reason given for not performing it. 4.5- No funnel plot or sensitivity analysis performed |  |
| Marto et al/ 2019 | Y | PY | Y | N |  | 2.2- No additional methods used (manual search/ref. search/ contacting authors/ searching institutional repositories) 4.2-Although the discussion mentions heterogeneity and small sample sizes as limitations, it does not explicitly justify the deviation from the planned meta-analysis outlined in the protocol. 4.5- No funnel plot or sensitivity analysis performed |  |
| Zhou et al/ 2021 | N | N | Y | PY |  | 1.1-No protocol developed, no registration (explicitly stated) 2.3-no keywords/MESH/search string for any database 4.2-They did not pre-register a protocol, so it is unclear whether the synthesis methods were fully pre-specified. |  |
| AlHabdan and AlAhmari/  2022 | Y | PN | PN | N |  | 2.2- No additional methods used (manual search/ref. search/ contacting authors/ searching institutional repositories) 2.5/3.1/3.5- No. of reviewers not stated 4.4- Heterogeneity not reported 4.5- No funnel plot or sensitivity analysis performed |  |
| Carneiro et al/ 2022 | N | Y | Y | N |  | 1.1-Protocol included: RCTs and quasi-RCTs. Review included only: RCTs. No justification or explanation was provided for excluding quasi-RCTs in the actual review. 4.5- No funnel plot or sensitivity analysis performed |  |
| Abdelkarim-Elafifi et al/ 2022 | N | N | N | N |  | 1.1- No Protocol  2.1- only two databases searched  2.2- No additional methods used (manual search/ref. search/ contacting authors/ searching institutional repositories) 3.4- RoB mentioned in methods but not reported in results/ discussion 4.4- Heterogeneity statistics not provided 4.5- No funnel plot or sensitivity analysis performed |  |
| Pion et al/ 2023 | N | N | Y | N |  | 1.1- In Protocol- databases to be searched were PubMed (MEDLINE), Scopus and Embase, and search strategy included RCT and retrospective study In final review- only PubMed (MEDLINE) is used without any explanation, only RCTs were included 2.1- only one database searched  2.2- No additional methods used (manual search/ref. search/ contacting authors/ searching institutional repositories) 4.5- No funnel plot or sensitivity analysis performed |  |
| Sgolastra et al/ 2011 | N | N | N | N |  | 1.1- No Protocol 1.2-inclusion criteria addressed effectiveness but did not explicitly include studies assessing safety, despite it being a stated secondary aim. 2.3-Full search strategy not given 2.5- One reviewer did study selection 3.1- One reviewer did data collection 3.5- One reviewer did quality assessment 4.4- Heterogeneity statistics not given 4.5- No funnel plot or sensitivity analysis performed |  |
| Oliveira da Rosa et al/ 2013 | N | N | N | N |  | 1.1- No Protocol 1.2-P and C elements of PICO not stated, Unclear question 2.3- Full search strategy not given 3.4/3.5/4.6- RoB not assessed 4.4- Heterogeneity statistics not given 4.5- No funnel plot or sensitivity analysis performed |  |
| Lin et al/ 2013 | N | N | N | N |  | 1.1- No Protocol 1.2-PICO not explicitly and clearly defined 2.3- Full search strategy not given 3.4/3.5/4.6- RoB not assessed 4.5- No funnel plot or sensitivity analysis performed |  |
| West et al/ 2015 | N | PY | N | N |  | 1.1-A protocol is said to have been designed, but it is not available, not published, and not registered 2.2- No additional methods used (manual search/ref. search/ contacting authors/ searching institutional repositories) 3.1/3.5- No. of reviewers not stated 4.4- Heterogeneity statistics not given 4.5- No funnel plot or sensitivity analysis performed |  |
| Rezazadeh et al/ 2019 | N | PN | N | N |  | 1.1- No Protocol  1.2-PICO not explicitly and not clearly defined 2.1- only two databases searched  2.2- No additional methods used (manual search/ref. search/ contacting authors/ searching institutional repositories) 2.3- no MESH term, no complete search strategy from any database. 3.4/3.5/4.6- RoB not assessed 4.4- Heterogeneity statistics not given 4.5- No funnel plot or sensitivity analysis performed |  |
| Shakeel et al/ 2022 | N | N | N | N |  | 1.1- No Protocol  1.2-PICO not explicitly and not clearly defined 2.2- No additional methods used (manual search/ref. search/ contacting authors/ searching institutional repositories) 2.3- no MESH term, no complete search strategy from any database. 2.5/3.1/3.5- No. of reviewers not stated 3.4/3.5/4.6- RoB not assessed 4.3-recommendations are stronger than the data justifies especially when- Only 6 studies included. Some showed no significant difference between groups. 4.4- Heterogeneity statistics not given 4.5- No funnel plot or sensitivity analysis performed |  |
| Lestari et al/ 2023 | N | N | N | N |  | 1.1- No Protocol  2.2- No additional methods used (manual search/ref. search/ contacting authors/ searching institutional repositories) 2.3- no MESH term, no complete search strategy from any database. 3.4/3.5/4.6- RoB not assessed 4.4- Heterogeneity statistics not given 4.5- No funnel plot or sensitivity analysis performed |  |
| Cerqueira et al/ 2025 | PY | PY | PY | N |  | 1.1- Reference search reported in Protocol but not done in  final review 2.2- No additional methods used (manual search/ref. search/ contacting authors/ searching institutional repositories) 2.4- Language restriction not reported in methodology although in Protocol they reported no language restriction. 3.5- No. of reviewers not stated 4.4- Heterogeneity statistics not given 4.5- No funnel plot or sensitivity analysis performed |  |
| Mohammadian et al/2025 | N | Y | Y | N |  | 1.1- No Protocol 4.4- Heterogeneity was not assessed 4.5- No funnel plot or sensitivity analysis performed |  |
|  |  |  |  |  |  |  | |
| **Colour coding for each item** | | **Yes(Y)/ Probably Yes (PY)** | **No information (NI)** | **Probably No/No** |  |  | |
| **Colour coding for overall risk of bias** | | **Low risk of bias** | **Unclear risk of bias** | **High risk of bias** |  |  | |
